# Mitochondrial transfer between BMSCs and Müller promotes mitochondrial fusion and suppresses gliosis in degenerative retina

**DOI:** 10.1016/j.isci.2024.110309

**Published:** 2024-06-20

**Authors:** Xiaona Huang, Luodan A, Hui Gao, Juncai He, Lingling Ge, Zhe Cha, Hong Gong, Xi Lin, Huiting Li, Yongping Tang, Dan Jiang, Xiaotang Fan, Haiwei Xu

**Affiliations:** 1 Southwest Eye Hospital, Southwest Hospital, Third Military Medical University (Army Medical University), Chongqing, China; 2 Key Lab of Visual Damage and Regeneration & Restoration of Chongqing, Chongqing, China; 3 Department of Military Cognitive Psychology, School of Psychology, Third Military Medical University (Army Medical University), Chongqing, China; 4 State Key Laboratory of Ophthalmology, Optometry and Visual Science, Eye Hospital, Wenzhou Medical University, Wenzhou 325027, China

**Keywords:** sensory neuroscience, cell biology

## Abstract

Mitochondrial dysfunction and Müller cells gliosis are significant pathological characteristics of retinal degeneration (RD) and causing blinding. Stem cell therapy is a promising treatment for RD, the recently accepted therapeutic mechanism is cell fusion induced materials transfer. However, whether materials including mitochondrial transfer between grafted stem cells and recipient’s cells contribute to suppressing gliosis and mechanism are unclear. In present study, we demonstrated that bone marrow mesenchymal stem cells (BMSCs) transferred mitochondria to Müller cells by cell fusion and tunneling nanotubes. BMSCs-derived mitochondria (BMSCs-mito) were integrated into mitochondrial network of Müller cells, improving mitochondrial function, reducing oxidative stress and gliosis, which protected visual function partially in the degenerative rat retina. RNA sequencing analysis revealed that BMSCs-mito increased mitochondrial DNA (mtDNA) content and facilitated mitochondrial fusion in damaged Müller cells. It suggests that mitochondrial transfer from BMSCs remodels Müller cells metabolism and suppresses gliosis; thus, delaying the degenerative progression of RD.

## Highlights

• Müller glia show mitochondria disorders in retinitis pigmentosa rats
• Mitochondrial transfer from BMSCs reduces oxidative stress of Müller glia
• BMSCs-mito reduce gliosis of Müller glia *in vivo* and *in vitro*
• BMSCs-mito promote mt-DNA and mitochondrial fusion to restore visual function

## Graphical abstract

**Figure:**
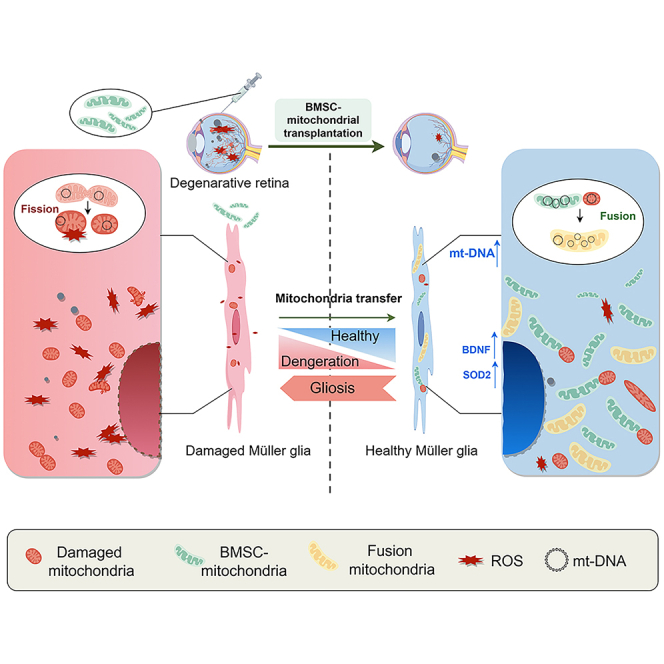

## Introduction

Retinal degeneration (RD) comprises a group of blinding eye diseases characterized by progressive degeneration and loss of retinal pigmented epithelial (RPE) cells and photoreceptors. Substantial reactive gliosis and excessive inflammatory responses are the pivotal pathological changes that occur during RD.^1^^,^^2^ RD is the leading cause of irreversible vision loss and blindness, without effective treatments currently available.

Müller cells are the predominant macroglia cell type in the retina.^3^ As supporting cells across the retina, Müller cells play key roles in retinal metabolism, anti-oxidative stress, and neuro-nutrition.^4^ Müller cells contribute to the maintenance of retinal homeostasis due to its metabolic symbiotic ability.^5^ Meanwhile, Müller cells are reprogrammed into progenitor cells and regenerate the whole retina in zebrafish during the retina injury, which have been regarded as endogenous stem cells,^6^ while in mammals, Müller cells usually form glial scar instead of reprogram, which usually aggravate the degenerative processes. Mitochondrial disorders have been identified as the initiating mechanism of gliosis in Müller cells which have been confirmed in diabetic retinopathy and age-related macular degeneration (AMD).^7^ As mitochondria and metabolism are pivotal regulators of cell fate, regulating mitochondrial dynamics and cell metabolism facilitates cell reprogramming.^8^^,^^9^ However, the role of mitochondria and energy metabolism in regulating the gliosis fate transition of Müller cells during RD is unclear.

Stem cell transplantation is a recognized treatment for RD with promising prospects.^10^ Recently, mounting evidence has demonstrated that grafted stem cells mainly transfer material to host cells through cell fusion; ultimately leading to a delay in RD.^11^^,^^12^^,^^13^ Among the multitype stem cell donors, bone mesenchymal stem cells (BMSCs) have the capacity for neuroprotection and immunoregulation in RD,^14^^,^^15^ with dozens of clinical trials being conducted in patients with RD^16^^,^^17^ because of their abundant sources, anti-inflammatory properties, and neurotrophic effects.^18^ Interestingly, endogenic bone marrow cells appear to preferentially fuse with Müller cells rather than other retinal cells.^19^ As the grafted stem cells exchange the material with the recipient’s cells through cell fusion, tunneling nanotubes (TNTs), or extracellular vesicles, the material transfer modes between transplanted BMSCs and Müller cells in the degenerative retina and underlying mechanisms have not been clarified so far.^20^

Mitochondria, as one of the most complex and important organelles, provide the necessary energy for cell activities. Mitochondrial dysfunction has been shown to be associated with many pathological changes and diseases. In the process of neurodegenerative diseases, mitochondrial fission, aging, mitochondrial DNA (mtDNA) deletion resulted in oxidative stress, and mitochondrial function loss.^21^ A growing body of literature has shown that mesenchymal stem cells (MSCs), idealized mitochondrial donors, can transfer mitochondria to somatic cells and improve mitochondrial function to rescue injured cells such as cardiomyocytes, pulmonary airway cells, and neurons.^22^^,^^23^^,^^24^^,^^25^ In the retina, intravitreal injection of induced pluripotent stem cell-derived MSCs transfer functional mitochondria to the damaged retinal ganglion, photoreceptor, and RPE cells to improve retinal function.^26^^,^^27^ We aimed to explore whether BMSCs transfer mitochondria to Müller cells to improve mitochondrial function and induce metabolic remodeling, how the mitochondria transfer influenced the cell fate of Müller cells and visual function of RD.

In the present study, we demonstrated the presence of mitochondrial disorders in the Müller cells of Royal College of Surgeons (RCS) rats, consistent with a previous report.^28^ In an established mitochondrial dysfunction cell model using the mitochondrial complex I (NADH: ubiquinone oxidoreductase) inhibitor rotenone (Müller-rot), which was cocultured with BMSCs or BMSC-derived mitochondria directly, we confirmed that mitochondrial transfer between BMSCs and Müller cells stabilized the mitochondrial membrane potential and reduced oxidative stress in rotenone-treated Müller cells. BMSCs-derived mitochondria (BMSCs-mito) were engulfed by Müller cells and integrated into their mitochondria, resulting in improved mitochondrial structure and function and reduced oxidative stress, apoptosis, and gliosis levels in rotenone-treated Müller cells. RNA-seq analysis showed that BMSCs-mito increased mtDNA content in the Müller cells. When transplanted into the retina of the RCS rats, BMSCs-mito was mainly taken up by the Müller cells, preserving visual function and reversing the gliotic fate of Müller cells. This suggests that mitochondrial transfer between BMSCs and injured Müller cells suppresses Müller cells gliosis by metabolic remodeling and promotes mitochondrial function; thus, delaying the process of RD.

## Results

### Mitochondrial transfer from BMSCs protected rotenone-induced damage of Müller cells

We extracted primary BMSCs and Müller cells as the object of study to explore the mitochondria transfer between these two cells. Primary Müller cells extracted from the retinas of Long-Evans (LE) rats at postnatal day (PND) 7 were determined by their expression of the specific markers, vimentin, glutamine synthetase (GS), and glial fibrillary acidic protein (GFAP) (Figure S1B). Primary BMSCs isolated from the bone marrow of LE rats at PND 28 showed a long, spindle-like morphology (Figure S1C1). BMSCs had the potential to differentiate into osteogenic (Figure S1C2), adaptogenic (Figure S1C3), and chondrogenic (Figure S1C4) cells. Flow cytometry showed that BMSCs primarily expressed classic markers, including CD90, CD44, and CD29, and were negative for CD45, CD34, and CD11b (Figure S1D), suggesting a high purity of BMSCs [29]. Müller cells were treated with the mitochondrial complex I inhibitor, rotenone at various concentrations (1, 5, and 25 μM) for 24 h to induce the mitochondrial dysfunction to mimic pathological changes of in retinal degenerative diseases. Rotenone caused cell shrinkage, nucleus swelling, and approximately 40% or 70% cell death at 5 or 25 μM, respectively (Figure S2A). Rotenone treatment caused that mitochondrial membrane potential (MMP) decreased, reactive oxygen species (ROS) increased, adenosine triphosphate (ATP) production and GS enzyme activity decreased in Müller cells in a concentration-dependent way (Figures S2B–S2E), which showed the mitochondrial dysfunction, oxidative stress, and the dynamic disorders of Müller cells. As 25 μM rotenone caused serious cell death, 5 μM rotenone treatment for 24 hours (h) was selected to induce mitochondrial damage in the subsequent experiments.

**Figure 1:**
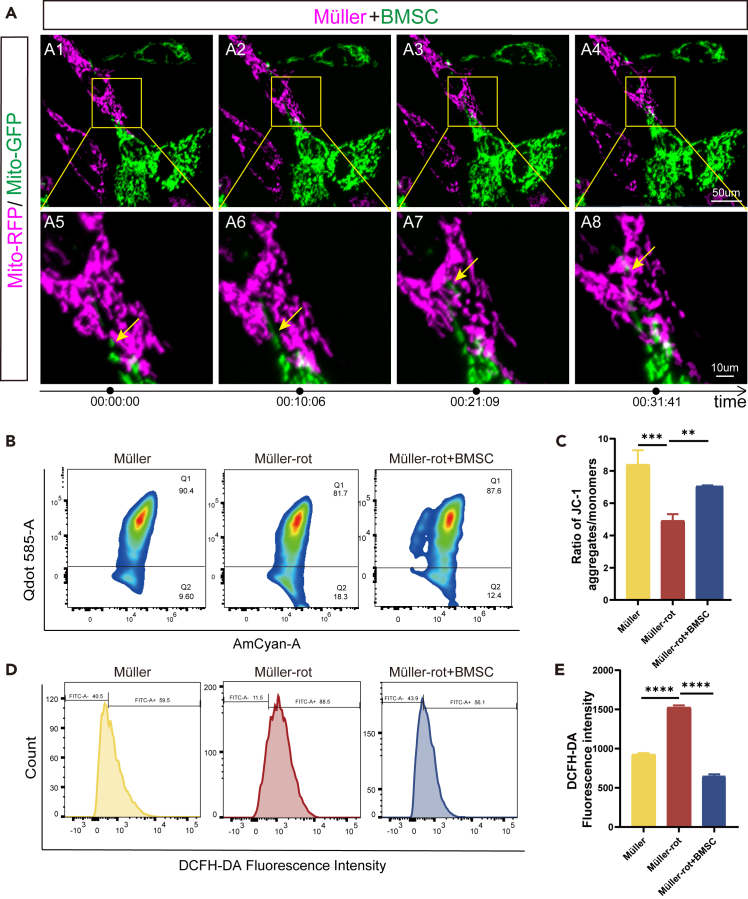
Mitochondria transfer occurred between cocultured bone marrow mesenchymal stem cells (BMSCs) and Müller cells and produced protection for rotenone-injured Müller (Müller-rot) cells (A) Dynamic process of mitochondrial transfer between Müller cells labeled with Mito-RFP (magenta) and BMSCs labeled with Mito-GFP (green) after direct coculture for 24 h. The phenomenon of increased mitochondrial network interaction between two cells has been observed. Yellow arrow points to the transferring mitochondria. (B) Coculture Müller-rot cells (treated with rotenone 5 μM for 24 h) with BMSCs. Flow cytometry analysis of mitochondrial membrane potential (MMP) using JC-1 in Müller-rot cells, and the influence of BMSCs coculture, with Müller cells as normal control groups. while Q1 (Qdot 585^+^) represents JC-1 aggregates: higher MMP, Q2 (Amcyan^+^) represents JC-1 monomers: lower MMP. Number below Qn is the ratio of this part. (C) Analysis of the ratio of MMP: JC-1 aggregates/JC-1 monomers. Higher ratio showed healthier mitochondrial function. *n* = 3. (D) Flow cytometry analysis of the intracellular reactive oxygen species (ROS) production in rotenone-injured Müller cells and the influences of BMSCs coculture for 24 h. (E) The relative fluorescence intensity of ROS production. *n* = 3. Data are presented as the mean ± standard deviation (SD), ∗∗*p* < 0.01; ∗∗∗*p* < 0.001; ∗∗∗∗*p* < 0.0001 (one-way analysis of variance [ANOVA] for C, E). Scale bars: 10 μm (A5-8), 50 μm (A1-A4).

To investigate whether BMSCs could transfer mitochondria to Müller cells, the Mito-COX8 protein was used for lentivirus packaging to label the mitochondria. Mitochondria of Müller cells were with the lentiviral-mitochondrial-red fluorescence protein (Mito-RFP); mitochondria of BMSCs were showed by the lentiviral-mitochondrial-green fluorescence protein (Mito-GFP). After the Mito-RFP and Mito-GFP both did well on mitochondria image formation, we conducted a direct co-culture of BMSCs and Müller cells in a 1:1 ratio. After co-culturing for 24 h, mitochondrial transfer between BMSCs and Müller cells was observed, and the exchange efficiency increased over time (Figure 1A). Cell fusion-mediated mitochondrial exchange was investigated by coculturing of celltrace violet labeled whole Müller cells with Mito-GFP labeled BMSCs (Figure S3A). Tunneling nanotube (TNT)-mediated mitochondrial exchange was investigated by coculturing of Mito-RFP labeled Müller cells and Mito-GFP labeled BMSCs (Figure S3B). The patterns of mitochondrial exchange observed in the present co-culture included cell fusion and TNT-mediated mitochondrial exchange (Figures S3A and S3B). Rotenone treatment-induced mitochondrial damage of Müller cells facilitated mitochondrial exchange between Müller cells and BMSCs. (Figures S3C and S3D). Dynamic real time imaging and three-dimensional (3D) reconstruction further confirmed the mitochondrial transfer (Videos S1 and S2). Flow cytometry analysis demonstrated that mitochondrial transfer reversed the reduction in MMP at a higher ratio of JC-1 aggregates/monometers, suggested the protection of mitochondrial function in rotenone-treated Müller cells (Figures 1B and 1C). At the same time, ROS levels showed that oxidative stress of Müller-rot cells was suppressed partially after cocultivation with BMSCs (Figures 1D and 1E). Therefore, mitochondrial exchange between BMSCs and Müller cells occurred via cell fusion and TNT, and cocultivation protected mitochondrial function and inhibited intracellular oxidative stress in rotenone-injured Müller cells.

Video S1. Dynamic real time imaging confirmed the mitochondrial transfer between BMSCs and Müller cells, related to Figure 1

Video S2. Three-dimensional (3D) reconstruction confirmed the mitochondrial transfer between BMSCs and Müller cells, related to Figure 1

### BMSCs-mito were fused with Müller cells and promoted the mitochondria fusion

**Figure 2:**
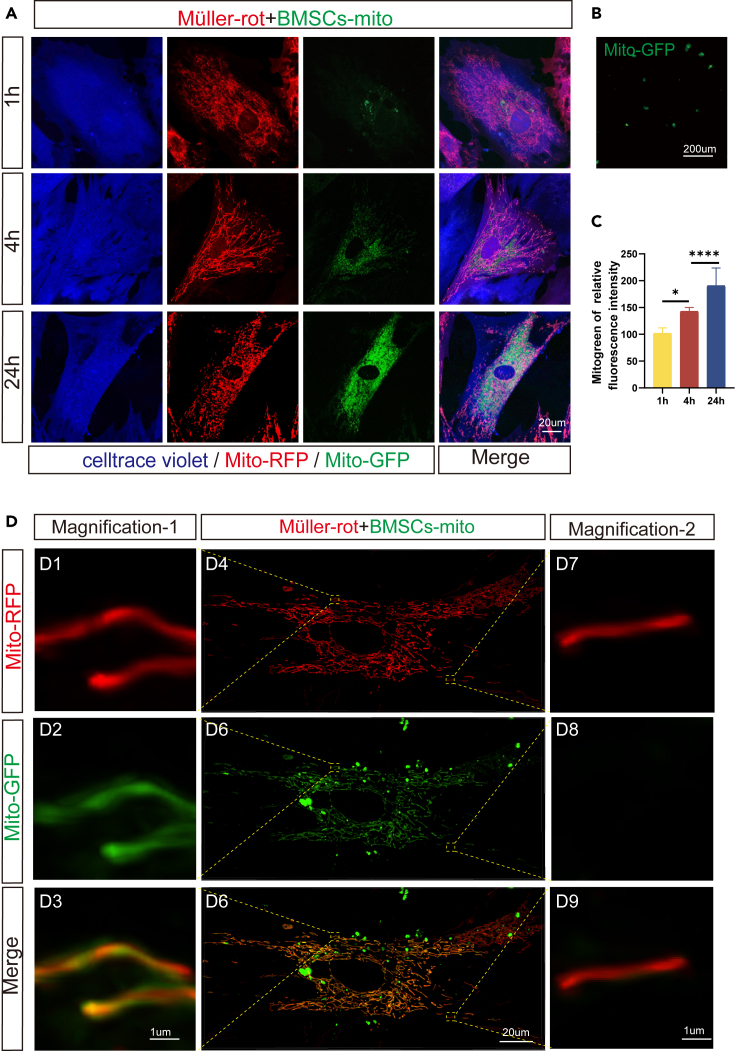
Isolated mitochondria from bone marrow mesenchymal stem cells (BMSCs-mito) were internalized and integrated into the mitochondrial network of Müller cells (A) Internalization of BMSCs-mito (Mito-GFP labeled) in the Müller cells treated with rotenone (Mito-RFP and celltrace violet-labeled) after coculture with isolated BMSCs-mito for 1, 4, and 24 h. Mito-GFP (green), Mito-RFP (red), celltrace violet (blue). (B) The representative images of the isolated mitochondria from BMSCs stained with Mito-GFP. (C) The relative fluorescence intensity analysis of BMSCs-mito internalized by rotenone-treated Müller after coculture with isolated BMSCs-mito for 24 h. *n* ≥ 5. (D) BMSCs-mito stained with Mito-GFP (green) was integrated into the mitochondrial network of Müller-rot cells labeled by Mito-RFP (red) after coculture for 24 h. D1-D3 shows the representative magnification of the mitochondrial integration and fusion of BMSCs-mito with the mitochondrial network of Müller-rot cells, whereas D7-D8 showed mitochondria of Müller-rot cells without integration with the BMSCs-mito. Data are presented as the mean ± standard deviation (SD). ∗*p* < 0.05; ∗∗∗∗*p* < 0.0001 (one-way analysis of variance [ANOVA] for C). Scale bars: 200 μm (B), 20 μm (A, D4, D5, D6), and 1 μm (D1-D3, D7-D9).

To further confirm the effect of BMSCs-mito, we labeled the BMSCs-mito with Mito-GFP, then, separated BMSCs-mito with a mitochondrial isolation kit (Figure 2B). Müller cells were labeled by Mito-RFP and celltrace violet, then were treated with 5 μM rotenone for 24 h (Müller-rot cells). Müller-rot cells were cocultured with BMSCs-mito at the ratio of BMSCs-mito from 5×10^6^ BMSCs/35 mm^2^ well for 1 h, 4 h, or 24 h. It showed that BMSCs-mito were internalized in Müller cells over time (Figures 2A and 2C). The internalization process was accelerated in the rotenone-treated Müller cells (Figures S4A and S4B). Interestingly, the internalized BMSCs-mito appeared to be distributed in the mitochondrial network of Müller cells at 24 h (Figures 2D4–2D6). High-resolution images showed that BMSCs-mito were co-localized with mitochondria of Müller cells, suggesting that parts of the BMSCs-mito were integrated with mitochondria of Müller cells (Figures 2D1–2D3), whereas some mitochondria of Müller cells failed to integrate with the BMSCs-mito in the mitochondrial network (Figures 2D7–2D9).

**Figure 3:**
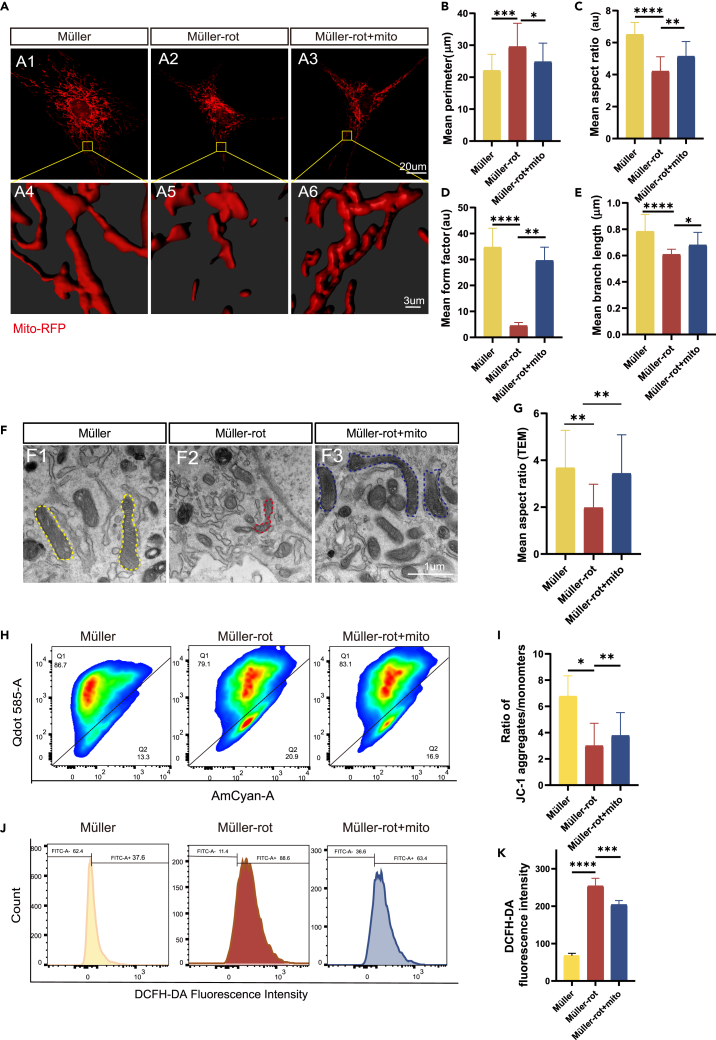
Mitochondrial morphology and function were enhanced in rotenone-treated Müller cells after coculturing with isolated mitochondria from bone marrow mesenchymal stem cells (BMSCs-mito) (A) 3D reconstruction images of mitochondrial morphology in Müller cells using imaris software. Müller and Müller-rot cells were labeled by Mito-RFP, then Müller-rot cells were cocultured with BMSCs-mito for 24 h. (B) Mean perimeter of mitochondria. *n* ≥ 25. (C) Mean aspect ratio of mitochondria. Aspect ratio is a measure of mitochondrial length; A value of 1 indicates a perfect circle and increases with elongated and elliptical mitochondria. *n* ≥ 20. (D) Mean form factor of mitochondria. The form factor is an indicator of mitochondrial length and degree of mitochondrial branching. A form factor value of 1 indicates round, unbranched mitochondria, while a higher FF value indicates longer, more branched mitochondria. *n* ≥ 24. (E) Mean branch length of mitochondria. *n* ≥ 24. (F) Transmission electron microscopy (TEM) images of mitochondria in different groups. During the group treated with rotenone, mitochondrial fragmentation and the mitochondrion swelled and became deformed, crest broke as the red dotted border, while the cocultivation with BMSCs-mito for 24 h enhanced the mitochondria fusion of Müller cells to a healthier mitochondrial morphology as the yellow or blue dotted border. (G) The analysis of the mean aspect ratio of mitochondria. *n* ≥ 10. (H) Flow cytometry analysis of mitochondrial membrane potential (MMP) by JC-1 in Müller cells injured by rotenone and the influence of BMSCs-mito coculture for 24 h, Q1 represented JC-1 aggregates: normal mitochondria, Q2 represented JC-1 monomers: MMP decreased. (I) The relative fluorescence intensity of MMP of the ratio of JC-1 aggregates and monomers. *n* = 3. (J) Flow cytometry analysis of the intracellular reactive oxygen species (ROS) production treated with BMSCs-mito coculture for 24 h after exposure of Müller cells to rotenone (rot). (K) The relative fluorescence intensity of ROS production in each group was analyzed using flow cytometry. *n* = 3. Data are presented as the mean ± standard deviation (SD). ∗*p* < 0.05; ∗∗*p* < 0.01; ∗∗∗*p* < 0.001; ∗∗∗∗*p* < 0.0001 (one-way analysis of variance [ANOVA] for B-E, G, I, K). Scale bars: 20 μm (A1-3), 3 μm (A4-6), and 1 μm (F).

Interestingly, both the integrated and non-integrated mitochondria in Müller cells exhibited an elongated and continuous morphology (Figure 2D). Then, we isolated unlabeled BMSCs-mito using mitochondrial isolation kit. 24 h after co-culture, investigated the effects of BMSCs-mito on mitochondrial morphology in rotenone treated Müller cells using three dimensional (3D) reconstruction with imaris software (Figure 3A). After treatment with rotenone, the mitochondria in Müller cells changed into a short and rounded morphology, suggesting the presence of mitochondrial fission, which tended to cause mitochondrial dysfunction (Figures 3A2 and 3A5). Mitochondria which were purified from BMSCs (BMSCs-mito) salvaged the fragmentation to a long tubular morphology like the normal Müller cells (Figures 3A3 and 3A6). Furthermore, quantitative changes of mitochondria morphology were analyzed by mitochondrial analyzer and MINA of the software ImageJ. Results showed that BMSCs-mito reversed the rotenone-induced increase in mitochondrial perimeter and the decrease in mitochondrial aspect ratio, form factor, and branch length, which matched the pictures (Figures 3B–3E). Besides, transmission electron microscopy (TEM) of Müller cells confirmed that BMSCs-mito improved mitochondrial fusion and cristae alterations in Müller cells (Figures 3F–3G). Therefore, BMSCs-mito was internalised by the mitochondria of Müller cells, thus enhanced the dynamics and mitochondrial morphology of rotenone-injured Müller cells.

### BMSCs-mito improved mitochondrial function and regulated the fate of Müller cells

**Figure 4:**
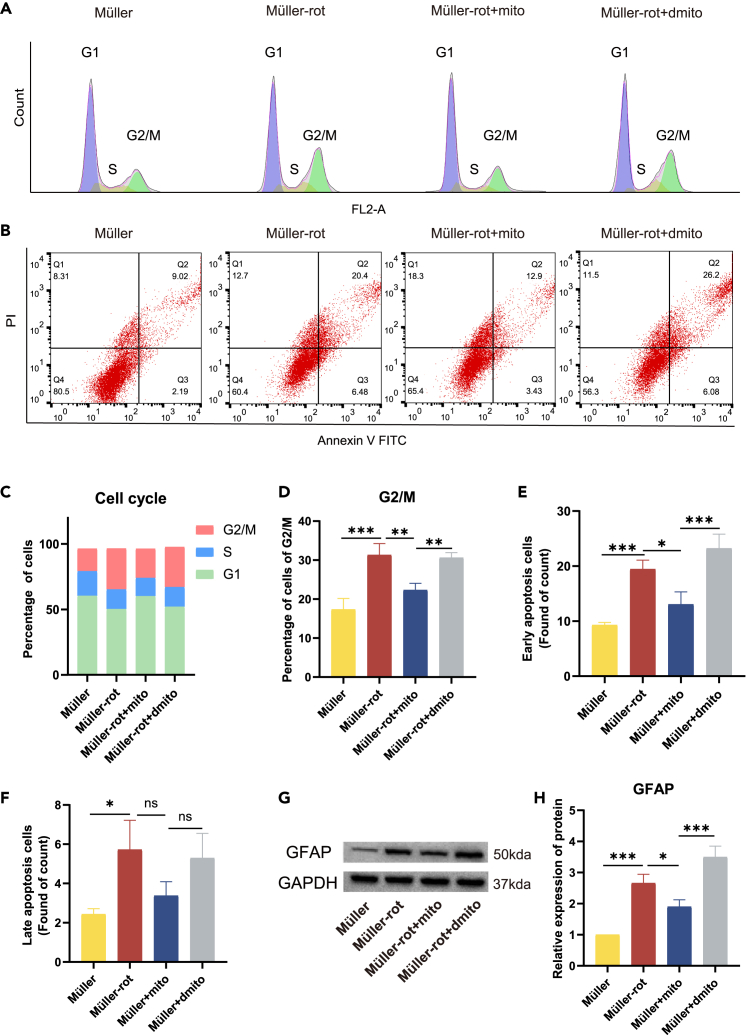
Effects of isolated mitochondria from bone marrow mesenchymal stem cells (BMSCs-mito) on the cell-cycle arrest and apoptosis rate in rotenone-injured Müller-rot cells (A) Cell cycle of normal Müller cells, Müller cells injured by rotenone (Müller-rot) and the influence of BMSCs-mito coculture. Müller-rot cells arrested at G2/M, BMSCs-mito internalization for 24 h changed the arrest. BMSCs-dmito were isolated from the BMSCs which treated by rotenone at the concentration of 0.5 μM for 24 h to get the mitochondrial dysfunction. (B) The flow analysis of apoptosis rate changed after coculture with BMSCs-mito for 24 h. Q2 showed the early apoptosis cells, Q3 showed the late apoptosis cells, and number below Q is the ratio of this type of cells (%). (C) Analysis of cell cycle. *n* = 3. (D) Analysis of G2/M of cell cycle. *n* = 3. (E) Count of early apoptosis. *n* = 3. (F) Count of late apoptosis. *n* = 3. (G) The representative images of the expression level of glial fibrillary acidic protein (GFAP) by western blot. (H) The ratio of GFAP/glyceraldehyde 3-phosphate dehydrogenase (GAPDH) in western blot, *n* = 3. Data are presented as the mean ± standard deviation (SD), ns, no significance; ∗*p* < 0.05; ∗∗*p* < 0.01; ∗∗∗*p* < 0.001; ∗∗∗∗*p* < 0.0001 (one-way analysis of variance [ANOVA] for D-F, H).

We also explored the influence of BMSCs-mito on mitochondrial function in Müller cells. After rotenone treatment, the treatment of BMSCs-mito for 24 h was used to rescue mitochondrial function in Müller cells. In consideration of the flow channel, BMSCs-mito and Müller cells were not labeled before cocultivation. Flow cytometric analysis of JC-1 staining showed that rotenone treatment decreased the MMP of Müller cells, whereas BMSCs-mito prevented this decline of MMP (Figures 3H and 3I). DCFA staining indicated that BMSCs-mito treatment reduced ROS production (Figures 3J and 3K). To verify the protection of BMSCs-mito, BMSCs were treated with 0.5 μM of rotenone for 24 h before BMSCs-mito were isolated (Figure S2F). After rotenone treatment, we isolated the damaged mitochondria from BMSCs, named as BMSCs-dmito. Cell cycle test showed that rotenone-induced mitochondrial damage caused G2/M cell-cycle arrest (Figures 4A, 4C, and 4D).^29^ The treatment of BMSCs-mito for 24 h decreased the percentage of Müller cells in the G2/M phase, rescued rotenone-induced cell-cycle arrest (Figures 4A, 4C, and 4D). Meanwhile, BMSCs-mito inhibited the increase in early and late apoptosis of Müller cells induced by rotenone (Figures 4B, 4E, and 4F). Western blot (WB) findings displayed that the GFAP expression in Müller cells increased following rotenone treatment, and BMSCs-mito alleviated gliosis in Müller cells (Figures 4G, 4H, S5A, and S5B). Altogether, BMSCs-mito improved mitochondrial dysfunction, cell-cycle arrest, and gliosis in Müller cells, while the damaged mitochondria of BMSCs had little protection.

### Müller cells in the retinas of RCS rats exhibited mitochondrial dysfunction and morphological abnormalities

RCS rats is a kind of autosomal recessive retinal pigmentosa animal model, which is a relatively mature animal model of RD first applied to the study of the cause and treatment of RP.^30^ As a classic animal model for the study of various types of RD, RCS has many similarities with human RP. Retinal photoreceptor cells in RCS rats developed PND 17. Rapid degeneration began PND 20. At PND 35, many photoreceptor cells were apoptotic. At PND 56, retinal pigment epithelial cells in the posterior pole began to lose. At PND 60, about 99% of the photoreceptor cells were degenerated. Around 3 months after birth, all photoreceptor cells disappear.^31^^,^^32^^,^^33^ As no mitochondrial damage in Müller cells of RCS rats has been reported previously, therefore, we explored this using MMP flow analysis and TEM for RCS retina which 7 weeks after birth (middle stage of the degenerative process), with the same age RDY rat as normal control groups. Single cell suspension dissociated from rat retinas by papain, then JC-1 staining of MMP was performed. After that, CD29 was incubated with retinal single cell suspension as a marker of Müller cells of the retina.^34^ Flow analysis displayed that CD29^+^ cells (Müller cells) made up about 5% of the cells in the retina (Figures S6A and S6C). Then, analysis the JC-1 of this group of CD29^+^ cells (Figure S6D). Results of JC-1 flow analysis confirmed the mitochondrial dysfunction of Müller cells in RCS rats compared with RDY rats (Figures S6E and S6F). Disruption of mitochondrial morphology in Müller cells was observed during RD in RCS rats, whereas mitochondria were elongated with prominent ridges of Müller cells in normal control rats (Figure S6G). The Müller cells in the retinas of RCS rats exhibited mitochondrial dysfunction and morphological abnormalities.

### BMSCs-mito transplantation reversed Müller cell gliosis and protected the visual function of RCS rats

**Figure 5:**
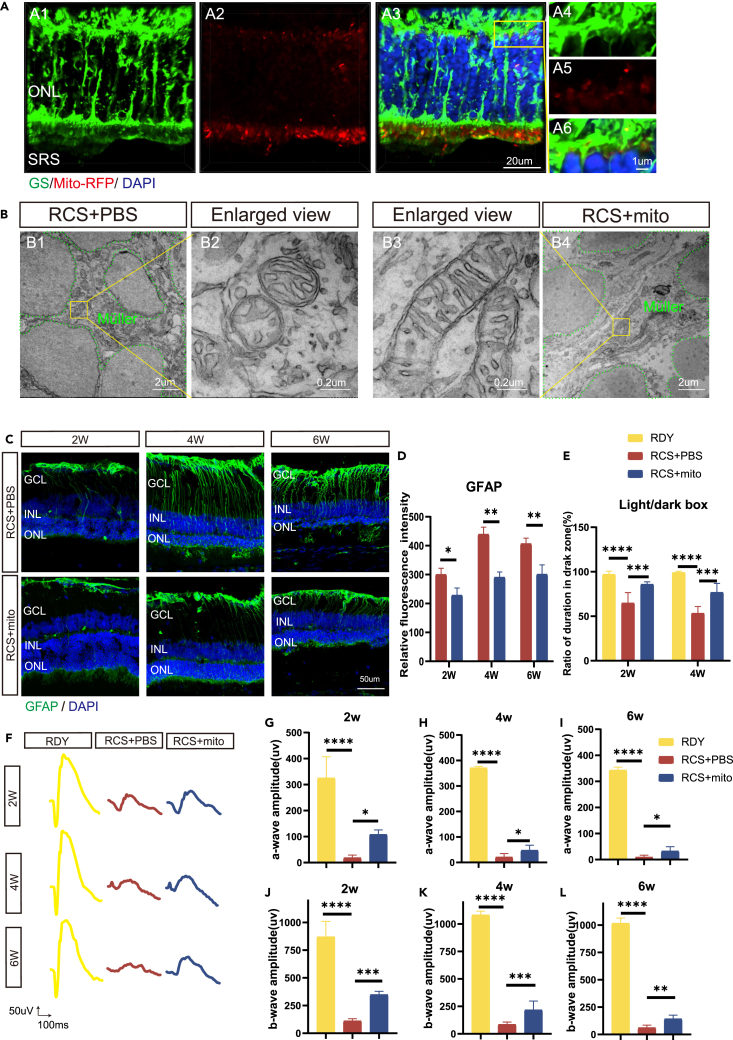
Isolated mitochondria from bone marrow mesenchymal stem cells (BMSCs-mito) were internalized by the retina and changed the gliosis of Müller cells and visual function in retina-degenerated Royal College of Surgeons (RCS) rats (A) BMSCs-mito labeled with Mito-RFP (red) was mainly internalized into the Müller cells stained with glutamine synthetase (GS) (green), outer nuclear layers (ONL), subretinal space (SRS). (B) Transmission electron microscopy results of mitochondria of Müller cells in RCS rat retina 4 weeks after transplantation of BMSCs-mito or phosphate-buffered saline (PBS). The green dotted line area represents Müller cells. In the RCS+PBS group, mitochondria underwent fission, whereas, in the group RCS+mito, mitochondria showed a preference for fusion, resulting in a healthier morphology. E2 is the magnification of E1, and E3 is the magnification of E4. (C) The representative images of the gliosis marker of Müller cells: Glial fibrillary acidic protein (GFAP) (green) and cell nuclear marker 4′,6-diamidino-2-phenylindole (DAPI) (blue) after BMSCs-mito transplantation at 2-, 4-, and 6 weeks. Ganglion cell layers (GCL), outer nuclear layers (ONL), inner nuclear layers (INL). (D) The relative fluorescence intensity of GFAP of B. n = 3. (E) Ratio of the duration in dark zones of rats in the light/dark box test in the normal rat groups (RDY), PBS transplantation into the RCS rat’s retina groups (RCS+PBS), BMSCs-mito transplantation into the RCS retina of RCS rats groups (RCS+mito). *n* = 6. (F) Representative image of electrophysiology at different time points: 2-, 4-, and 6 weeks after BMSCs-mito transplantation. (G–I) The protection of BMSCs-mito transplantation groups compared to PBS groups and RDY rats of the a-wave amplitude at 2-, 4-, and 6 weeks after transplantation. *n* = 6. (J–L) The protection of BMSCs-mito transplantation groups on b-wave amplitude compared to RCS+PBS and RDY groups at 2-, 4-, and 6 weeks after transplantation. *n* = 6. Data are presented as the mean ± standard deviation (SD), ∗*p* < 0.05; ∗∗*p* < 0.01; ∗∗∗*p* < 0.001; ∗∗∗∗*p* < 0.0001 (one-way analysis of variance [ANOVA] for E, G-L ttest for D). Scale bars: 20 μm (A1-A3); 1 μm (A4-A6); 2 μm (B1, E4); 0.2 μm (B2, B3); 50 μm(C).

To assess the therapeutic effect of BMSCs-mito on RD, the BMSCs-mito was transplanted into the subretinal space (SRS) of RCS rats 3 weeks after birth (initial stage of degenerative process), with the same volume of PBS as the control. As for the safety of subretinal transplantation, we checked the puzzle pictures of whole retina of RCS rats at 6 weeks after transplantation. Results showed that detachment occurred in the transplant area, tumor formation was not found (Figures S7A–S7C). To reduce immune rejection, oral cyclosporine A dissolved in drinking water was administered to all animals starting 24 h prior to transplantation and continuing for two weeks following the procedure. To find the location of BMSCs-mito, we use the Mito-RFP to label the BMSCs, and then isolated the mitochondria of BMSCs to get the BMSCs-mito (Mito-RFP). Three days after mitochondrial transplantation, the majority of Mito-RFP labeled BMSCs-mito were observed in the SRS, and a small portion of BMSCs-mito was adjacent to the process of Müller cells (Figure S8A). Most of the BMSCs-mito into the retina were co-labeled with GS, indicating that BMSCs-mito were internalized by Müller cells mainly (Figure 5A). Some BMSCs-mito moved into RPE cells labeled by RPE65, which closed to the SRS of retina (Figure S8B). Few BMSCs-mito located into photoreceptor cells labeled by Rhodopsin, but interestingly, a large portion of BMSCs-mito gathered into the Müller cells around photoreceptors and foot process of Müller cells closed to SRS (Figures 5A and S8C). BMSCs-mito transplantation resulted in changes in mitochondrial morphology from a short and rounded morphology in the degenerative retina to a long tubular morphology, which presented healthier mitochondria (Figure 5B). Müller cells underwent excessive gliosis and formed a GFAP positive glial scar at the outer limitation membrane of RCS rats, whereas grafted BMSCs-mito suppressed gliosis of Müller cells at 2-, 4-, and 6 weeks after BMSCs-mito transplantation (Figures 5C and 5D). TUNEL staining showed that apoptosis of RCS retina occurred almost at outer nuclear layers and BMSCs-mito transplantation suppressed this apoptosis (Figures S8D and S8E). The thickness of the outer nuclear layer of the retina was protected after the transplantation of mitochondria (Figures S8F and S8G). Light/box test showed BMSCs-mito increased the time into drak box of RCS rats, which presented a healthier visual function (Figure 5E). Electroretinograms (ERG) was used to determine the visual function of RCS rats with BMSCs-mito transplantation, and representative waveform plots in PBS and BMSCs-mito are shown (Figure 5F). When compared to the PBS group, the a- and b-wave amplitudes in BMSCs-mito group increased significantly (Figures 5G–5L). Thus, we demonstrated that BMSCs-mito was internalized by the Müller cells of the degenerative retina, alleviated gliosis of Müller cells, and protected the visual function of RCS rats.

### BMSCs-mito regulated gene expression pattern of electron transport chain between mitochondria and nucleus

**Figure 6:**
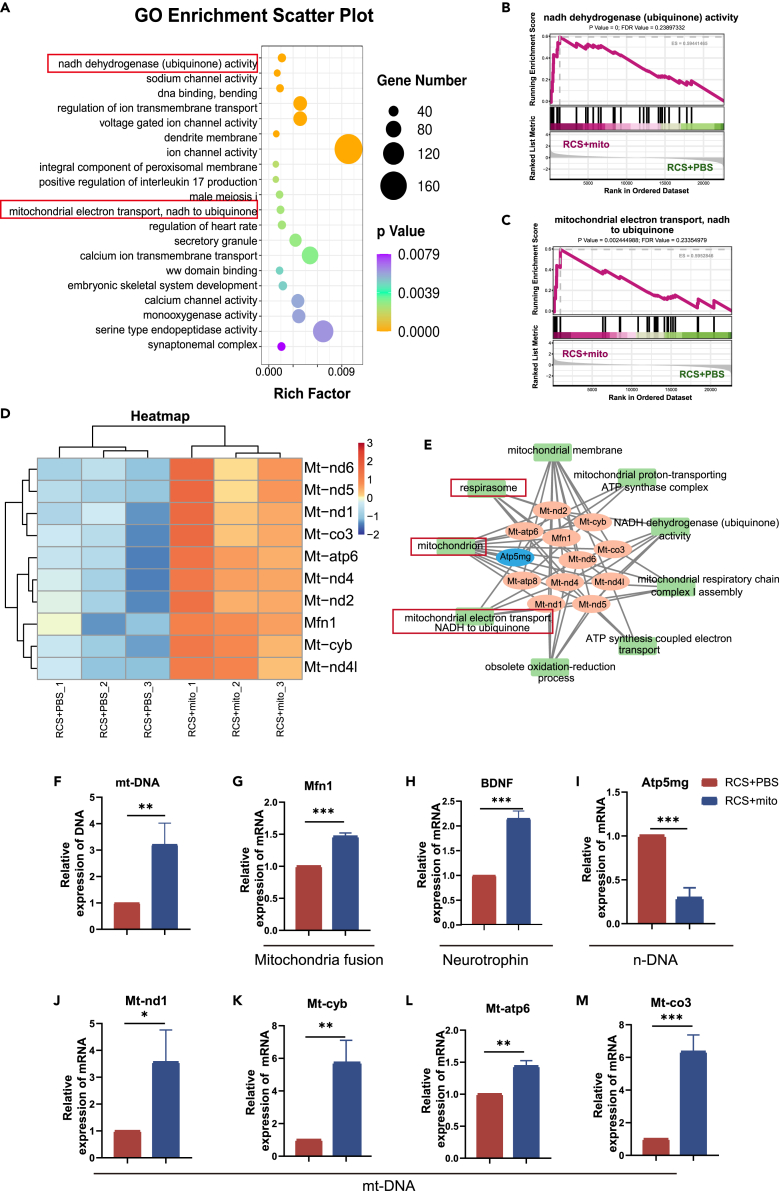
Gene ontology (GO) of gene set enrichment analysis (GSEA) of differentially expressed genes involved in the protection of isolated mitochondria from bone marrow mesenchymal stem cells (BMSCs-mito) in Royal College of Surgeons (RCS) rats *in vivo* (A) The top 20 most significantly changed GO terms in the GO enrichment analyses of the BMSCs-mito transplanted into the RCS rats (RCS+mito) compared to the phosphate-buffered saline (PBS) transplanted into the RCS rats (RCS+PBS) group. (B and C) Significantly up-expressed gene set in RCS+mito groups compared to RCS+PBS groups by the GO of GSEA. (D) The differentially expressed messenger ribonucleic acids (mRNAs) in RCS+mito and RCS+PBS groups. Each row represents a single mRNA, and each column represents one tissue sample. Red denoted high relative expression, and blue indicated low relative expression. (E) GO term analysis for differently expressed mRNAs. (F) Mitochondrial deoxyribonucleic acid (mtDNA) expression. (G) *Mfn1* mRNA expression. (H) *BDNF* mRNA expression. (I) *Atp5mg* mRNA expression. (J) *Mt-nd1* mRNA expression. (K) *Mt-cyb* mRNA expression. (L) *Mt-atp6* mRNA expression. (M) *Mt-co3* mRNA expression. Data are presented as the mean ± standard deviation (SD), ∗*p* < 0.05; ∗∗*p* < 0.01; ∗∗∗*p* < 0.001; (test for F–M). F–M: *n* = 3.

To elucidate the process by which BMSCs-mito protects Müller cells and degenerative retinas, we performed RNA-seq of the retinas 4 weeks after BMSCs-mito transplantation (Table S3). A principal-component analysis (PCA) plot based on all levels of gene expression showed good consistency within groups (Figure S9A). A comparison between PBS and BMSCs-mito indicated that 833 upregulated and 1,393 downregulated differentially expressed genes (DEGs) were identified in the BMSCs-mito group (Figures S9B–S9D). All DEGs were included in the GO gene function classification system of GSEA, and the top 20 GO pathways revealed that mitochondrial structure and function (NADH dehydrogenase activity and mitochondrial electron transport pathways) played a prominent role (Figure 6A). Both pathways were consistently upregulated in the BMSCs-mito groups (Figures 6B and 6C). Based on these results, we demonstrated that mitochondria were particularly important mediators in protecting BMSCs-mito.

Furthermore, gene expression of the electron transport chain (ETC) encoded by mtDNA (*Mt-nd1*, *Mt-nd2*, *Mt-nd4*, *Mt-nd4l*, *Mt-nd5*, *Mt-nd6*, *Mt-co3*, *Mt-atp6*, and *Mt-atp8*) was upregulated in BMSCs-mito, whereas that of the nuclear DNA gene (*Atp5mg*) was significantly downregulated after transplantation of BMSCs-mito into the degenerative retina. The interaction between genes and GO pathways exerted a widespread influence on the mitochondrial ETC, oxidation-reduction, and ATP levels (Figures 6D, 6E, and S9F).^35^ Notably, the expression of *Mfn1*, a protein that modulated the content of mtDNA and promoted mitochondrial fusion, was markedly increased in the BMSCs-mito group (Figures 6D and 6E).^36^^,^^37^ Furthermore, BMSCs-mito significantly increased the *Mfn1* and mtDNA content and related genes and markedly decreased the nuclear DNA in the mitochondrial chain in the PCR test (Figure 6F–6M).

Interestingly, the interaction of all DEGs showed a strong relationship with the following pathways: mitochondrial biogenesis, retinal development, glutamate receptor, and calcium channel activity, which were connected through the brain-derived neurotrophic factor (*BDNF*) gene, a key neurotrophic factor secreted by Müller cells in the retina^38^ (Figures S9E–S9I).

**Figure 7:**
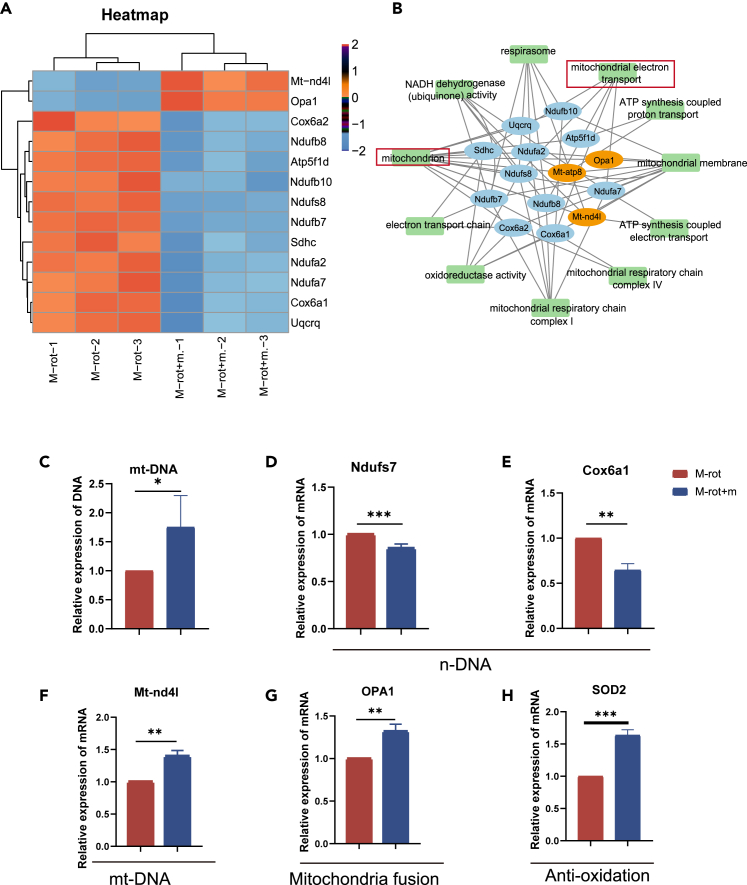
Heatmap and analysis of differentially expressed genes involved in the protection of isolated mitochondria from bone marrow mesenchymal stem cells (BMSCs-mito) cocultured with Müller-rot cells *in vitro* (A) Heatmap of the differentially expressed messenger ribonucleic acids (mRNAs) in Müller-rot+mito (M-rot+m) and Müller-rot (M-rot) groups. Each row represents a single mRNA, and each column represents one tissue sample. Red represented high relative expression, and blue represented low relative expression. (B) Interaction diagram gene ontology (GO) pathway and differential genes. (C) Mitochondrial deoxyribonucleic acid (mtDNA) expression analysis. (D) *Ndufs7* mRNA expression. (E) *Cox6a1* mRNA expression. (F) *Mt-nd4l* mRNA expression. (G) *OPA1* mRNA expression. (H) *SOD2* mRNA expression. Data are presented as the mean ± standard deviation (SD), ∗p < 0.05; ∗∗*p* < 0.01; ∗∗∗*p* < 0.001; (t test for C–H). C–H: *n* = 3.

Subsequently, we explored whether changes in the mtDNA content and mitochondrial biogenesis occurred in Müller cells *in vitro* (Table S4). Müller-rot cells (M-rot) and Müller-rot cells + mito (M-rot+m) groups were collected for RNA extraction. PCA, DEGs, volcano plot, and heatmaps of all genes showed good clustering (Figures S10A–S10D). Consistent with our hypothesis, the gene expression of mitochondrial ETC changed significantly (Figure 7A). The expression of mtDNA (*Mt-nd4l*, *Mt-atp8*) increased markedly, whereas that of nDNA (*cox6a1*, *cox6a2*, *nudfs7*, *atp5f1d*) decreased significantly in the M-rot+m group compared with M-rot group (Figure 7A). The pathways of interaction of these genes focused on the mitochondrial ETC, oxidation, and ATP, consistent with that of the *in vivo* results (Figure 7B). Superoxide dismutase (*SOD2*) is a potent mitochondrial antioxidant in neurodegenerative diseases,^39^ which increased (Figure 7H). The expression of the mitochondrial fusion gene *OPA1* also increased in M-rot+m group (Figure 7G). The changes of mtDNA, *Ndufs7*, *cox6a1*, and *M*t-*nd4l* levels were verified at the DNA and RNA levels (Figures 7C–7F). Thus, we speculated that BMSCs-mito regulated mitochondrial function and mtDNA content by accelerating mitochondrial fusion and *BDNF* levels in Müller cells; thereby, delayed the retinal degenerative process of RD diseases.

## Discussion

In this work, we proved that injured Müller cells received mitochondrial transfers from BMSCs, which remodeled metabolism and inhibited gliosis to delay RD. Mitochondrial transfer from BMSCs to the injured Müller cells enhanced mitochondrial function, suppressed oxidative stress, and inhibited gliosis *in vitro*. After transplantation of BMSCs-mito into the SRS of RCS rats, BMSCs-mito were mainly engulfed by Müller cells, which reversed mitochondrial impairment, gliosis, and RD. RNA-seq revealed that there was a noticeable rise in mtDNA and mitochondrial fusion gene levels.

RD is a multifactorial disease with a complicated pathogenesis linked to various genetic mutations and environmental factors.^1^^,^^40^^,^^41^ Mitochondrial damage and dysfunction during RD development are considered as major pathological changes in retinal cells.^42^ Besides energy production, mitochondria are major organelles that regulate cell survival and death, calcium ion homeostasis, ROS production, and oxidative stress.^43^^,^^44^ In the previous studies, mitochondrial damage has been shown to cause apoptosis in the retinal ganglion cells, RPE cells, and photoreceptors.^42^^,^^45^ Hutto et al. showed that mitochondrial stress in cone photoreceptors triggers the selective movement of damaged mitochondria so that these damaged pyramidal mitochondria can be exported to glial cells, where they undergo degradation.^46^ However, few studies have demonstrated the role of mitochondrial damage in Müller cells and its influence on cell fate determination.^7^ In the retina of a woman with macular telangiectasia type 2, mitochondrial swelling, reduced number of cristae, and abnormal accumulation of electron-dense substances were observed in Müller cells.^47^ Single-cell RNA-seq has revealed that a portion of Müller cells from patients with AMD showed reduced mtDNA expression.^28^ In a mouse model of glaucoma, Müller cells exhibited mitochondrial damage.^48^ In this study, we confirmed that mitochondrial damage in Müller cells in the retina of RCS rats included mitochondrial fragmentation, absence of mitochondrial cristate, lower MMP, and reduced mtDNA content and expression, indicating that mitochondrial impairment may be involved in Müller cells gliosis during RD.

Moreover, it has been believed that transplanted stem cells protect the injured retina mainly through cell fusion-induced material transfer.^20^ It was confirmed that grafted BMSCs fused with Müller cells, induced the reprogramming of the Müller cells, and produced neuroprotection of the degenerated retina.^19^ However, the categories of transferred materials, patterns of transfer, and underlying mechanisms need to be explored. By direct co-culture of BMSCs and Müller cells, we showed that cell fusion between BMSCs and Müller cells induced mitochondrial transfer. TNT is vital for mitochondrial transfer. This is consistent with previous studies in which MSCs transferred mitochondria to injured cardiomyocytes, pulmonary airway cells, and neuronal cells, improved the cell survival ratio, and enhanced regeneration.^24^ In the present study, we observed that the mitochondria transferred from BMSCs were partially integrated into the mitochondrial network of Müller cells, which improved mitochondrial function, reshaped metabolism, and contributed to the survival and gliosis inhibition of Müller cells. Surely, we think that the effect of retinal protection is partly due to the Müller cells, as the retina is a complex network among kinds of cells, the influence is multitype and needs to be further explored. This study provides a possible way to the mitochondria therapy on retina degeneration diseases.

Mitochondrial transfer therapy has been explored in the retina diseases characterized by mitochondria dysfunction.^26^^,^^27^ Isolated mitochondria from stem cells have shown a significant therapeutic effect on spinal cord^49^ and optic nerve injuries.^50^ Recent research injected mitochondria of RCS rat liver into intravitreal space of RCS rat retina, which protects the retinal thickness and VEP.^51^ We directly extracted mitochondria from BMSCs and transplanted them into the sub-retinal space of RCS rats, also protected the retina thickness and ERGs, demonstrating that the grafted mitochondria markedly delayed RD, which was consistent with previous reports. Besides, the mitochondria location and further influence and mechanisms were explored in our study.

Mitochondrial dynamics, such as fusion and fission, affect mitochondrial function.^52^ Mitochondria can clear or rescue damaged mitochondrial function via mitochondria fusion.^53^ It is reported that mitochondrial fusion protects vision against retinal ischemia/reperfusion.^54^ The electronic respiratory chain is the basic structure for mitochondrial function. Two sets of coding systems coexist, with mtDNA primarily comprising essential structural components, and nuclear DNA encoding the remaining supplementary components and a few critical structures.^55^ The content and homeostasis of mtDNA are important because they affect mitochondrial biogenesis and function.^56^^,^^57^ Thus, repairing mitochondrial DNA is essential for maintaining cell function.^58^ RNA-seq showed that the mitochondria-related gene pathway was highly enriched, the mitochondrial fusion gene MFN1 and the mitochondrial electron respiratory chain-related gene increased, the nuclear DNA decreased, while the mtDNA content increased. Further, *in vitro* experiments and Müller cells RNA-seq also showed the similar gene changes in mtDNA, demonstrating that mitochondrial transfer enhances the function of retinal cells by accelerating mitochondrial fusion and increasing mtDNA content.

Additionally, we found that *BDNF* was significantly increased in the retina of BMSCs-mito transplanted RCS rats, and the genes associated with calcium ion channels, glutamate receptor channels, mitochondrial biogenesis, and retinal development-related genes such as *Pax6* and *Sox2* were involved in mitochondrial transfer-induced retinal protection. As *BDNF* regulates glutamate metabolism,^59^ promotes mtDNA,^60^ nourishes nerves,^61^ and inhibits oxidation,^62^ elevated *BDNF* expression in the degenerated retina produces neuroprotection. Mitochondrial transplantation protects against astrocytic *BDNF* and promotes neuronal survival in mice with traumatic brain injury.^63^ Mitochondria transplantation into primary mouse neurons showed neuroprotective effects.^64^ Müller cells played key roles in metabolic symbiosis, oxidative stress, endogenous stem cells, and nutritional support.^65^

Mitochondrial transfer from the BMSC protects the injured retina by promoting mitochondrial fusion and mtDNA content in Müller cells which rescues the function of mitochondria and suppresses the gliosis. This study provides a new method for the stem cell treatment of RD and proposes additional possibilities for improving mitochondrial disorders.

### Limitations of the study

Four pathways facilitate mitochondrial transfer between stem cells and other cells: TNT, cell fusion, vesicles, and gap junctions.^66^ Only TNT and cell fusion were observed in this study. The details of the mitochondrial exchange pathways and their influencing factors need to be further explored. Moreover, mitochondrial transfer between BMSCs and Müller cells was observed *in vitro* while faileded to be confirmed *in vivo*, which was a limitation of this technique. As Müller cells predominantly engulf mitochondria, the exchange and influences of mitochondria between grafted BMSCs and other retinal cells is not deeply understood. In future studies, we will explore mitochondrial-based therapy and its influencing factors in RD treatment.

## STAR★Methods

### Key resources table

**Table:** 

REAGENT or RESOURCE	SOURCE	IDENTIFIER
**Antibodies**		

Glial fibrillary protein (GFAP)	Abcam	Cat# ab7260; RRID: AB_305808
Glutamine synthetase (GS)	ABclonal	ABclonal Cat# A19641; RRID: AB_2862711
GAPDH	ABclonal	Cat#AC033; RRID: AB_2769570
RPE65	Santa cruz	Cat#SC390787; RRID: AB_3105798
Rhodopsin	Abcam	Cat#AB98887; RRID: AB_10696805
Vimentin	Santa cruz	Cat#SC6260; RRID: AB_628437
488 donkey-mouse	Thermo Fisher Scientific	Cat# A-21202; RRID: AB_141607
568 donkey-rabbit	Thermo Fisher Scientific	Cat#A10042; RRID: AB_2534017
488 donkey-rabbit	Thermo Fisher Scientific	Cat#A21206; RRID: AB_2535792
HRP-Goat anti-rabbit	Beyotime	Cat#A0208; RRID: AB_2892644
HRP-Goat anti-mouse	Beyotime	Cat#A0216; RRID: AB_2860575

**Bacterial and virus strains**		

Mito-GFP	Gene	Cat#LV-71293
Mito-RFP	Gene	Cat#LV-71294

**Chemicals, peptides, and recombinant proteins**		

medium for rat BMSCs	OriCell	Cat#RAXMX-90011
Celltrace violet	Invitrogen	Cat#C34557
Rotenone	Sigma	Cat#R8875
Phosphate buffer saline (PBS)	HyClone	Cat#SH30256.LS
DMSO	Sigma-Aldrich	Cat#D8418
TRIzol reagent	Invitrogen	Cat#15596026
BSA	Sangon biotech	Cat#A602440-0050
Penicillin/streptomycin	Gibco	Cat#15140122
FBS	Gbico	Cat#SH3007
DMEM	Pricella	Cat#PM150210

**Critical commercial assays**		

mitochondrial isolation kit	Thermo Fisher	Cat#89874
ATP assay	Beyotime	Cat#S0027
CCK-8 assay	Beyotime	Cat#C0038
Glutamine synthetase activity assay	Solarbio	Cat#BC0910
JC-1 kit	Beyotime	Cat#C2003S
Apoptosis assay	Beyotime	Cat#C1062L
gDNA extraction kit	TIANGEN	Cat#DP304-02
TUNEL	Beyotime	Cat#C1089
PrimeScript™ RT reagent Kit	Takara	Cat#RR037A

**Deposited data**		

RNA-seq data *in vitro*	This paper	SRA database PRJNA1113168
RNA-seq data *in vivo*	This paper	SRA database PRJNA1113189

**Experimental models: Organisms/strains**		

Royal College of Surgeons (RCS) rats	Third Military Medical University	N/A
RDY rats	Third Military Medical University	N/A
LE rats	Third Military Medical University	N/A

**Oligonucleotides**		

Primers, see Table S2	This paper	N/A

**Software and algorithms**		

ImageJ	NIH	https://imagej.net/ij/; RRID: SCR_003070
GraphPad Prism	Graphpad Software Inc.	https://www.graphpad.com/scientific-software/prism/; RRID: SCR_002798
FlowJo	BD Biosciences	https://www.flowjo.com/; RRID: SCR_008520
Imaris	OXFORD	https://imaris.oxinst.com/; RRID: SCR_00737

### Resource availability

#### Lead contact

Further information and requests for resources and reagents should be directed to and will be fulfilled by the lead contact, Haiwei xu (xuhaiwei@tmmu.edu.cn).

#### Materials availability

This study did not generate new unique reagents.

#### Data and code availability

RNA-seq data reported in this paper have been shared in the SRA database publicly accessible, under the accession number were SRA: PRJNA1113168, SRA: PRJNA1113189.

This paper does not report original code.

Any additional information required to reanalyze the data reported in this work paper is available from the lead contact upon request.

### Experimental model and study participant details

#### Primary Müller glia cells culture

Müller cells were harvested from the eyes of Long-Evans (LE) rats at PND 7, and the enrichment process was carried out as per previously published procedures.^3^^,^^67^ To purify the population of cells, the target cells were gathered, separated, and grown in DMEM with 20% FBS for five days. The third passage of primary isolated Müller cells was identified by immunofluorescence staining for vimentin, glial fibrillary acidic protein (GFAP) as well as glutamine synthetase (GS) (Table S1).

#### Isolation of bone marrow and BMSCs cell culture

Following the centrifugation of tibia and femora from LE rats at PND 28, bone marrow was extracted as a modified previous method.^68^ The isolated BMSCs were cultured with the complete medium for rat BMSCs (RAXMX-90011, OriCell). Subsequent experiments utilized cells from passages 3 to 5. BMSCs were characterized via these cell surface markers: CD11b, CD29, CD34, CD44, CD45, and CD90 (Dakewe). Chondrogenic, lipogenic and osteogenic induced multilineage differentiation of BMSCs was carried out *in vitro* (OriCell).

#### Animals and ethical statement

PND 21 or 49 were employed for Royal College of Surgeons (RCS) rats together with their normal control (RDY) rats, and PND 7 or 28 for LE rats. The rats of both sexes were raised at the Experimental Animal Centre of the Third Military Medical University (Army Medical University) in a pathogen-free environment with a 12-hour cycle of light and dark and unrestricted access to water and food. All tissue collection along with the experimental process was carried out in accordance with a protocol authorized by the Institutional Review Board of the Third Military Medical University (Army Medical University) (No. AMUWEC20182139).

### Method details

#### Cell labeling and mitochondrial tracking

Mitochondria morphologies were labeled using Lentiviral-Mitochondrial-Green Fluorescence Protein (Mito-GFP) (LV-71293, Gene) and Lentiviral-Mitochondrial-Red Fluorescence Protein (Mito-RFP) (LV-71294, Gene).^69^ The prepared Mito-GFP or Mito-RFP plasmid was used for lentivirus packaging, and the Mito-COX8-GFP or RFP lentivirus was transfected into the mitochondria of cells. Celltrace violet (C34557, Invitrogen) labeling was applied to show the whole cell in blue at 405 nm using both flow cytometry and confocal laser scanning microscopy.^26^

#### Establishing mitochondrial dysfunction *in vitro* model of Müller cells and BMSCs

Pre-treatment of Müller cells or BMSCs was conducted with mitochondrial complex I inhibitor rotenone (R8875, Sigma) for 24 h under different concentrations at 50–60% cell density to establish a mitochondrial dysfunction cell model.^70^ For Müller cells, we chose the concentration of 5 μM rotenone treating for 24 h as the suitable protocol for *in vitro* experiments. For BMSCs, the concentration of 0.5 μM rotenone treated for 24 h gets the ideal damage.

#### Coculture of Müller cells and BMSCs

After 24 h, the rotenone treated Müller cells (Müller-rot cells) were rinsed three times utilizing PBS. Subsequently, the BMSCs and Müller-rot cells were collected and mixed at a 1:1 ratio in a culture medium, which consisted of a complete medium for rat BMSCs (with 20% FBS) and a complete medium for Müller cells. Cells were cultivated at 5 × 10^4^ cells/cm^2^ for one day in a cell culture flask (Corning).^71^ Müller-rot and Müller cells were used as controls without cocultivation.

#### Assessment of mitochondrial transfer

Müller cells were labeled with Mito-RFP and celltrace violet, and BMSCs were labeled with Mito-GFP before cocultivation.^72^ After 24 h cocultivation, mitochondria transfer patterns between Müller cells and BMSCs were assayed via adopting a live cell imaging system (IX83, OLYMPUS) or confocal microscope (LSM880, ZEISS). Analysis the typical mitochondrial transfer cells (with the ways of cell fusion or tunneling nanotubes). The ratio of mitochondrial transfer was analyzed as the number of mitochondrial exchanging Müller cells in number of all Müller cells in the field.

#### Isolation of mitochondria from BMSCs and coculture with Müller cells

Prior to separation, the BMSCs mitochondria (BMSCs-mito) were labelled utilizing Mito-GFP. Using a mitochondrial isolation kit (89874; Thermo Fisher), mitochondria were extracted from the BMSCs.^49^ In a nutshell, BMSCs were extracted by centrifuging at 850 × g for 2 minutes (min) and later removing the supernatant. The BMSCs were placed in an ice-filled tube and given Mitochondrial Isolation Reagent A. After 5 seconds of medium speed vertexing, the tube was left to incubate on ice for 2 min. Mitochondrial isolation reagent B was added, and the tube was vortexed 5 times at its fastest speed and placed on ice for 5 min. Then mitochondria isolation reagent C was added, and the tube was repeatedly inverted to mix it well, and it was centrifuged at 700 × g and a temperature for 10 minutes. After centrifuging for 15 min at 12,000 × g, the mitochondria were collected, and the supernatant was transferred to a fresh tube. Isolated mitochondria from the rotenone damaged BMSCs named BMSCs-dmito.

To explore the efficiencies of internalization, Mito-RFP was utilized to label the mitochondria of Müller-rot cells, celltrace violet was utilized to mark those mitochondria, and Mito-GFP was utilized to stain the mitochondria of BMSCs. After that, Müller-rot cells were co-cultured with mitochondria (from 5×10^6^ BMSCs/ 35 mm well) and examined at various intervals (1-, 4-, or 24 h). Müller groups, Müller-rot cells groups, and Müller-rot cells cocultured with BMSCs-dmito groups were used as controls. Pictures were captured by Nikon AX or LSM880, ZEISS.

#### Adenosine triphosphate measurement glutamine synthetase activity assay

Applying an adenosine triphosphate (ATP) assay kit (S0027, Beyotime), the amount of ATP was ascertained.^49^ Following collection of Müller cells treated with rotenone (0-, 1-, 5-, and 25 μM), they were lysed in lysis buffer (100 μL) on ice; lysate was gathered and centrifuged at 12,000 × g for 3 min. Each supernatant was divided into 20 μL wells in an opaque 96-well plate, each with 100 μL of ATP detection working dilution. Thermo Varioskan Flash was exploited to measure luminescence.

#### Cell viability measurement

CCK-8 (C0038, Beyotime) was applied for the determination of cell viability.^73^ Müller cells, subjected to 24 h treatment with 0-, 1-, 5-, and 25 μM rotenone, were gathered. They were later rinsed with PBS thrice before being incubated with DMEM supplemented with a 10% CCK-8 assay solution at 37°C for 60 min. At 450 nm, optical density was determined with a microplate reader (Thermo Varioskan Flash).

#### 3D reconstruction of mitochondria in Müller cells

Image multiple continuous layers pictures of the Müller cells (labelled by Mito-RFP) using Nikon AX or LSM880, ZEISS. 3D reconstruction was done using the surface rendering of software imaris.^74^

#### Glutamine synthetase activity assay

GS was determined using a GS Kit (BC0910, Solarbio), as previously reported.^75^ Müller cells were harvested and put in a centrifuge tube after being exposed to rotenone (0-, 1-, 5-, and 25 μM) for one day. Following a 5-min centrifugation at 750 g at 4 °C, the supernatant was discarded. For every five million cells, extract (1 mL) was also added. After the cells were broken up by ultrasonic vibrations, they were centrifuged for 10 min at 8000 g and a temperature of 4 °C. The supernatant was gathered, and at 540 nm, the absorbance was identified through spectrophotometer (Infinite F50, Switzerland) for GS activity.

#### Reactive oxygen species assay

Reactive oxygen species (ROS) were identified in Müller cells by means of 2,7-Dichlorodihydrofluorescein diacetate (DCFH-DA) assay (Beyotime).^73^ Müller cells were treated with DCFH-DA for half an hour at 37 °C with a dose of 10 μM, following the manufacturer's instructions. Using a confocal laser-scanning microscope (LSM 880, Zeiss), images were captured. The flow cytometry analysis was performed to determine the ROS levels in living cells using flow cytometry (BD FACS Calibur), utilizing fluorescein 5-isothiocyanate (FITC) fluorescent channel and excitation light of 488 nm.

#### Mitochondrial membrane potential assay

Müller cells variations in mitochondrial membrane potential (MMP) were measured with a JC-1 kit (C2003S, Beyotime). JC-1 is an ideal fluorescent probe for detecting ΔΨm of mitochondrial membrane potential, which can detect cells, tissues, or purification Mitochondrial membrane potential. The decrease of mitochondrial membrane potential means the dysfunction of mitochondrial function. At high mitochondrial membrane potential, JC-1 accumulates in the matrix of the mitochondria, forming a polymer (JC-1 aggregates) that produces red fluorescence light; When the mitochondrial membrane potential is low, JC-1 cannot accumulate in the matrix of the mitochondria, and JC-1 is a monomer (JC-1 monomers), which can produce green fluorescence. In this way, it is very convenient to detect the change of mitochondrial membrane potential by the change of fluorescence color. The ratio of the JC-1 aggregates / JC-1 monomers can present the MMP level, which shows the function of mitochondria. The proportion of mitochondrial depolarization is often measured by the relative ratio of red or green fluorescence. To make new JC-1 staining solutions, JC-1 stock solutions were diluted with buffer 200 times in accordance with the instructions of the manufacturer. After 30 min of staining solution incubation at 37°C in the absence of light, the Müller cells were rinsed three times utilizing JC-1 buffer. To take photographs, a confocal laser scanning microscope (LSM 880, Zeiss) was employed. The procedure of cell treatment for flow cytometry analysis was referred to formerly described methods. Via exploiting flow cytometry (BD FACS Calibur) with an excitation light of 405 nm and the fluorescent channel Qdot-585 (JC-1 aggregates) and Amycan (JC-1 monomer), the levels of living cell JC-1 were ascertained.

#### Apoptosis assay

After being exposed to rotenone for one day, Müller cells were cultivated with separated BMSCs-mito (5 × 10^6^ BMSCs/30 mm well) for another 24 h. The apoptosis rate was examined through a Beyotime annexin V-FITC/PI apoptosis kit. Müller cells were treated with binding buffer (500 μL) after being twice rinsed with cold PBS at the end of the incubation time. Following the addition of PI (10 μL) and Annexin V-FITC (5 μL), cells were subjected to FACS analysis. Early necrotic cells were classified as PI-negative and annexin V-positive cells, whereas late necrotic cells were thought to be PI-negative and annexin V-negative cells.

#### Flow of retinal muller cell dissociation and identification and MMP

Collected animal retinas on ice, three retinas put into a 2.0 mL EP tube with 1 mL papain (13.5 U/ml) (Worthington), 37 degrees, shake with 180 RPM at 37 °C for 4 min. Blow Gently, strain with a 40 μm strainer to get the retinal single-cell suspension. After centrifuge, the supernatant was removed, the cells were re-suspended, and incubated with mitochondrial membrane potential (MMP) on the process of JC-1 kit (Hyclone). After the JC-1 staining, incubated at 4 °C for 15 min in PBS containing anti-CD29 (Clone Ha2/5, BD Biosciences, Heidelberg, Germany).^34^^,^^76^ The cells were washed with PBS once and detected by flow cytometry.

#### Electroretinography analysis

At 2-, 4-, and 6-weeks following transplantation, electroretinograms (ERG) were taken. The electroretinographic activity was assessed in compliance with accepted practices.^77^ The rats were acclimated to the dark overnight prior to the ERG testing, and all tests were carried out in low red light. Rats were given 1% sodium pentobarbital for anaesthesia, and 1% tropicamide for pupil dilation. To get the ERG signals, a reference electrode and a clip-on contact lens electrode were applied to the tongue and cornea, separately. Various light stimulus intensities were applied to record the amplitudes of the a- and b-waves. The electrophysiological equipment we employed in these tests was a Reti-Scan System (Roland, Germany) and a Stimulator LS-100/200 (Mayo Corporation, Aichi, Japan). 3.0 cd.s.m2 spot joint rod-cone responses were the data that were proposed in this investigation.

#### Mitochondrial transplantation

According to earlier approaches, RCS rats at PND 21 were employed for mitochondrial transplantation into the SRS.^67^ Pentobarbital injections intraperitoneally were utilized for anaesthetizing RCS rats. Tropicam (5 mg/mL) was employed to dilate the pupils. BMSCs-mito (separated from 1 × 10^7^ BMSCs) was suspected in PBS (3 μL) and PBS of the same volume was used as a control, they were gradually injected into the SRS of the temporal retina approximately 2.0 mm from the optic disc via a small scleral incision utilizing a microinjector (10 μL). The corneal puncture was implemented to lower the intraocular pressure and stop cell export. A small retinal detachment was visible via the dilated pupil following injection. Oral cyclosporine A (210 mg/l; Sandoz, Camberley) dissolved in drinking water was administered to all animals starting 24 h prior to transplantation and continuing for two weeks following the procedure.

#### Transmission electron microscopy

Following experimentation, the rats were subjected to euthanasia, and the eyes were rapidly extracted and immersed in 2.5% glutaraldehyde for 1 h. The retina-choroid-sclera complex was then isolated and sliced into small pieces, each about 1 mm³ in size, which were exposed to 2.5% glutaraldehyde at 4 °C for one day. Cultured Müller cells were collected via cytocentrifugation and treated with 2.5% glutaraldehyde at 4 °C for one day. After three rinses with phosphate buffer (0.1 M, pH 7.0), samples were fixed for 1 to 2 h in a 1% osmic acid solution. They were then rinsed three more times with phosphate buffer (0.1 M, pH 7.0). After that, the samples were dried with a gradient ethanol solution. The cells or sections were treated with pure acetone for 20 min after a 20 min treatment with 100% ethanol, and they were later implanted for 1 h. The infiltrating samples were implanted and heated at a temperature of 70 °C for the duration of an overnight treatment with a pure embedding agent. Samples were sliced into 70-90 nm slices via an ultrathin slicing machine, and they were dyed for 5-10 min with a 50% ethanol-saturated uranium acetate solution and lead citrate solution, separately. The samples were examined using a TEM (HITACHI HT7800) after drying.

#### Western blot analysis

Müller cells were lysed in a buffer with 1% protease inhibitors after being washed twice in ice-cold PBS. A portion of the total protein was separated using aliquots on SDS-PAGE precast gels (Nanjing ACE Biotechnology) at 160 V for 20 min. The protein was then quickly transferred to a polyvinylidene difluoride membrane (PVDF) membrane for 25 min at 400 mA. Membranes were cultivated with the primary antibodies, including GFAP and glyceraldehyde 3-phosphate dehydrogenase (GAPDH), overnight at 4 °C after being blocked for 20 min at 25 °C through a rapid blocking solution. The next day, the samples were rinsed three times in tris-buffered saline with 0.1% tween-20 (TBST) and incubated with the secondary antibodies for an hour at 25 °C (Table S1). Protein band signals were identified via applying an enhanced chemiluminescence (ECL) luminescence kit (Mengbio) and visualized with a ChemiDoc Imaging system (Bio-Rad). Lastly, ImageJ (1.42, National Institutes of Health) was employed for analyzing the gray value for each band.

#### Light/dark box test

The light/dark box test is a traditional test for visual ethology, with advantage of the rat's darkening.^78^ The box was 45 cm∗27 cm∗27 cm, light zone part of box is 2/3, while the dark zone part is 1/3. These two zones are connected with a hole(7.5 cm∗7.5 cm). Wipe the box with alcohol and dry naturally, insert the baffle of the passage between the light and dark room. After 12 h of dark acclimation, the rats were placed in the dark zone, and acclimated for 3 min. The baffle between the light and dark room was extracted, so that the rats could move freely in both the light and dark room. The light in the open room was turned on and the rat's activity was recorded for 5 min, and the time of the rats in the black box was counted.

#### Quantitative polymerase chain reaction

Utilizing TRIzol reagent (TaKaRa, Japan) as per the instructions of the manufacturer, extraction of total RNA was conducted from the retinas of three mice from diverse groups to analyze the mRNA levels of target genes. Then, via the Primer Script RT reagent kit and a genomic DNA (gDNA) eraser (TaKaRa, Japan), deoxyribonucleic acid (cDNA) was created from the RNA. Sequences for the gene-specific primer pairs are displayed in Table S2. TB Green was employed in conjunction with RT-PCR detection equipment (CTF96; Bio-Rad, USA) to identify the amplification products of cDNA samples. Using a 2^−ΔΔCT^ method, the target genes’ expression levels were determined in relation to the control gene, beta [β]-actin.

#### Quantification of mitochondrial DNA (mtDNA) content

The mtDNA content was measured using quantitative RT-PCR (qRT-PCR) and normalized to nuclear DNA amplification. We extracted DNA using the plant gDNA extraction kit (DP304-02; TIANGEN) as per instructions of the manufacturer.^79^ qPCR was implemented on gDNA via exploiting SYBR Premix Ex Taq in triplicate for each sample. Primer pair sequences for mtDNA are shown in Table S2.^80^ With the 2^−ΔΔCT^ approach, the mtDNA relative expression was determined, and normalized to β-actin.

#### Immunofluorescence staining

RCS rats' eyes were enucleated, and they were subsequently fixed for 30 min at 4 °C in 4% PFA (HyClone).^77^ Following corneal and lens removal applying a microscope (Olympus, Tokyo, Japan), the tissue was infiltrated with 30% sucrose for an overnight stewing at 4 °C after being preserved in PFA for 1.5 h. After removing the lens and cornea and separating the eyes from their orbits, the eyecups were separated. Subsequently, the tissues were transferred to an ideal cutting temperature solution (Sakura Finetek, USA) at a temperature of -80 °C, and a freezing microtome (Thermo Fisher Scientific, Waltham, USA) was employed to cut them into sagittal slices with a thickness of 14 μm. To prepare the cells, they were seeded onto slides and fixed for 15 min at 4 °C using 4% PFA. The sections were produced via the cultured with GFAP, 0.25% BSA (Sigma-Aldrich), and primary antibodies in PBS at 4 °C overnight. This was done after the sections were triple-washed in PBS and treated with 3% BSA and 0.3% Triton X-100 solution for half an hour at 37 °C. After that, the sections underwent three 5-min washes before being left to be incubated at room temperature for one hour with the secondary antibody. TUNEL labeled apoptosis cells, were stained by the protocol of the kit (HyClone). Following washing, and 4',6-diamidino-2-phenylindole (DAPI) was utilized as a counterstain for the nuclei before the sections were mounted. With the use of a confocal laser scanning microscope (LSM 880, Zeiss), slices or slides were photographed.

#### RNA-Seq and bioinformatic analysis

Retinal tissues were collected at 4 weeks after transplantation with PBS or BMSCs-mito. To facilitate this, RCS rats treated with vehicle PBS and BMSCs-mito were designated as the RCS+PBS and RCS+mito groups, separately. For each set of samples that were examined, three bio-replicates were employed. Dynabeads Oligo (dT) was exploited to separate the mRNA from the total RNA after extraction. Short fragments of mRNA were obtained after purification. After cleaving the RNA fragments, the cDNA libraries were created with reverse transcription, resulting in a mean insert size of 300 ± 50 bp. Illumina NovaSeq 6000 (LC-BioTechnology Co., Ltd., Hangzhou, China) was exploited to conduct the 2×150 bp paired-end sequencing (PE150). The FastQC program was adopted to confirm the sequence quality. Through the HISAT2 package, the reads from each sample were aligned to the research species' reference genome. Each sample's mapped reads were put together, and the ultimate transcriptomes from every sample were produced. To quantify expression abundance for mRNA, all levels of transcript expression were evaluated, and fragments per kilobase of transcript per million mapped reads (FPKM) were computed. The DESeq2 software was utilized to perform the analysis of differential gene expression through the imputing of read count data matrices. The thresholds utilized to select differentially expressed genes (DEGs) were p-value <0.05 and fold changes ≥1.2. Patterns of DEG were visualized as heat maps with clusters. The raised genes (p <0.05 and FC ≥1.2) and decreased genes (p <0.05 and FC ≤-1.2) were represented in red and blue, respectively. According to the size of the normalized enrichment score (NES), the 20 most significant GO terms in the GSEA were selected.

*In vitro*, we collected Müller-rot cells (M-rot) and Müller-rot cocultured with BMSCs-mito (M-rot+m). DEGs were chosen according to p-value <0.05 and fold changes ≥1.2 as the thresholds and details described above. Bioinformatic analysis was performed using the OmicStudio tools at https://www.omicstudio.cn/tool or Majorbio tools at https://www.majorbio.com/.

### Quantification and statistical analysis

#### Statistical analysis

Data are expressed as the mean ± SD from a minimum of three biological samples. Applying GraphPad Prism 9.2.0, a one-way ANOVA with Tukey's multiple comparison test and Benjamini-Hochberg correction were carried out for multiple comparisons and multiple t-tests, resp. At p < 0.05, statistical significance was established.

## Supplemental information

Document S1. Figures S1–S10 and Tables S1 and S2

Table S3. RNA-seq data about DEGs of the retinas 4 weeks after BMSCs-mito transplantation, related to Figure 6

Table S4. RNA-seq data about DEGs in Müller cells *in vitro*, related to Figure 7
