# Supplementary material for: Mitochondrial transfer between BMSCs and Müller promotes mitochondrial fusion and suppresses gliosis in degenerative retina
Source: iScience. 2024 Jun 20;27(7):110309. doi: 10.1016/j.isci.2024.110309 (PMC11269791; doi:10.1016/j.isci.2024.110309)
Supplement: Document S1. Figures S1–S10 and Tables S1 and S2 [file mmc1.pdf]

iScience, Volume *27*

Supplemental information

Mitochondrial transfer between BMSCs

and Müller promotes mitochondrial fusion

and suppresses gliosis in degenerative retina

Xiaona Huang, Luodan A, Hui Gao, Juncai He, Lingling Ge, Zhe Cha, Hong Gong, Xi Lin, Huiting Li, Yongping Tang, Dan Jiang, Xiaotang Fan, and Haiwei Xu

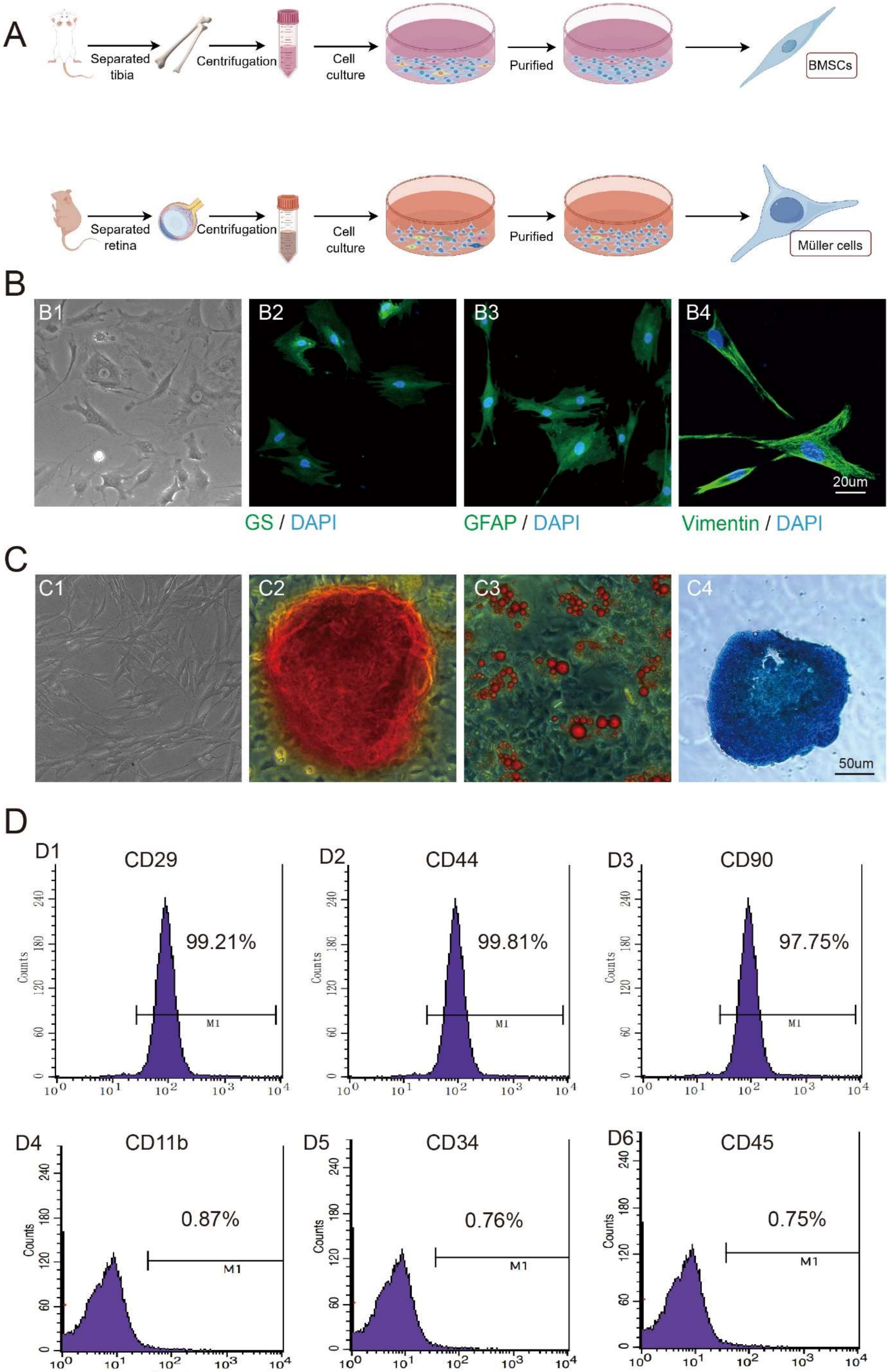

Figure S1. Identification of primary Müller cells and BMSCs of SD rat, related to STAR Methods.

(A) Experments design.

(B) The representative identification images of specific markers of Müller cells. B1 white phase of Müller cells; B2 GS (green) and DAPI (blue); B3 GFAP (green) and DAPI (blue); A4 vimentin(green) and DAPI (blue).

(C) The representative identification images of specific markers of BMSCs. C1 white phase of BMSCs; C2 osteogenic differentiation of BMSCs; C3 Adipogenic differentiation of BMSCs; C4 Chondrogenic differentiation of BMSCs.

(D) Positive and negative antibody of BMSCs identification by flow cytometry. D1-D3 Positive antibody; D4-D6 negative antibody.

Scale bars: 20μm (B); 50μm (C).

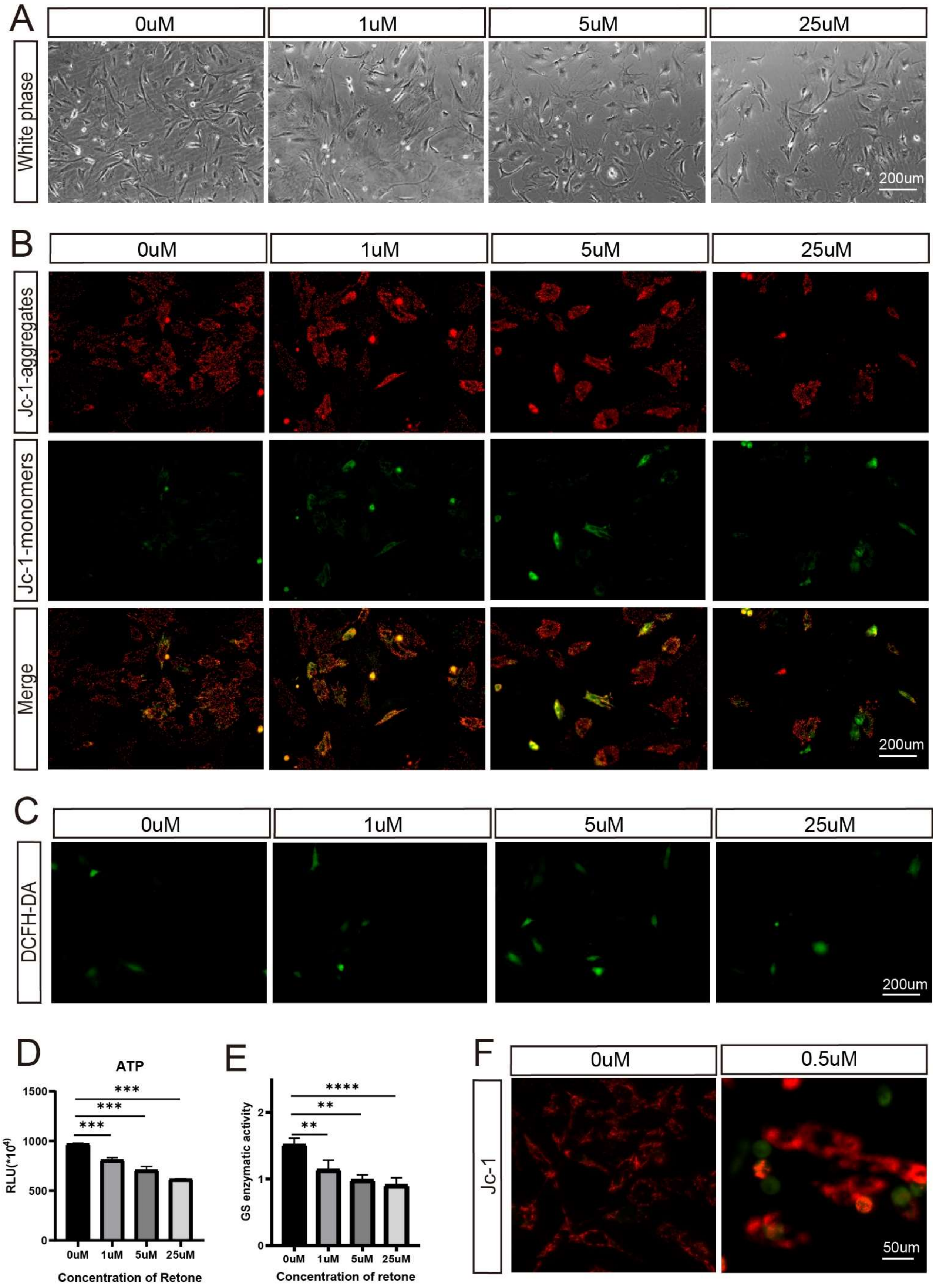

Figure S2. The influence of Rotenone-Induced Model of mitochondrial damage to Müller cells or BMSCs, related to Figure 1.

(A) White phase of Müller cells treated under different rotenone concentrations from 0μM to 25μM.

(B) Imaging mitochondrial membrane potentials of Müller cells. Normal mitochondria are fluorescently labelled in red, and the fluorescence changes from green to red when the MMP decreases.

(C) Exhibition of ROS (Reactive Oxygen Species) of Müller cells.

(D) Statistical analysis figure of ATP level in Müller cells. n=3.

(E) Analysis of the glutamine synthetase activity in Müller cells. n=4.

(F) Imaging mitochondrial membrane potentials of BMSCs cells of the treatment of rotenone at the concentrations of 0 or 0.5uM for 24h.

Data are presented as the mean ± SD, *P < 0.05; **P < 0.01; ***P < 0.001; ****P < 0.0001 (one-way ANOVA for D, E). Scale bars: 200μm (A, B, C). 50μm (H).

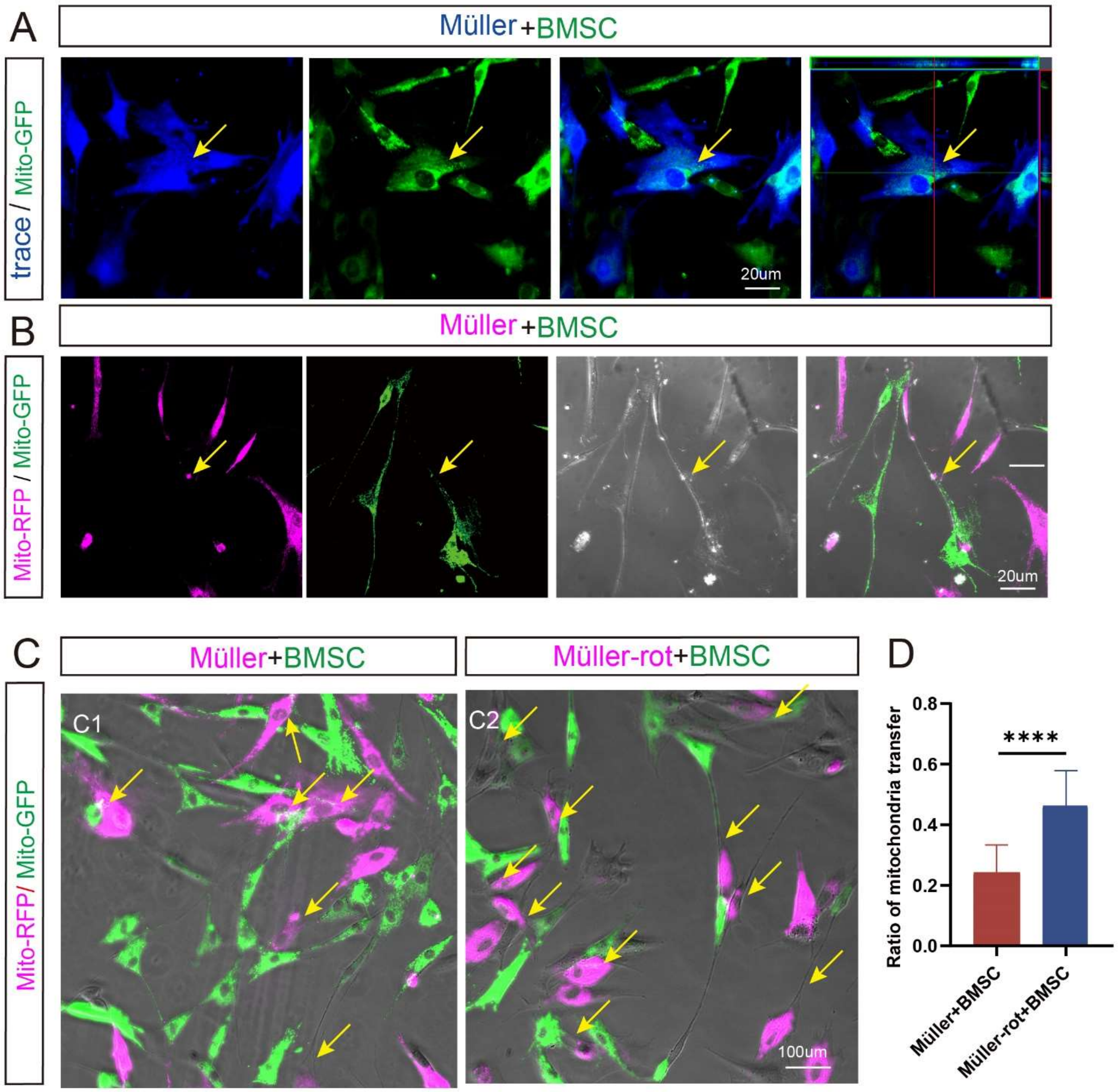

Figure S3. Ways of mitochondria transfer occur during direct co-culture of BMSCs and Müller-rot cells, related to Figure 1.

(A) Ways of mitochondria transfer: cell fusion; Müller cells labelled by cell trace (blue); BMSCs labelled by Mito-GFP (green). Yellow arrows pointed to the transferred mitochondria.

(B) Ways of mitochondria transfer: TNT (tunnelling nanotubes) after direct co-culture 24h between BMSCs with Müller-rot cells; Müller cells labelled by Mitot-RFP (magenta), BMSCs labelled by Mito-GFP (green). Yellow dotted lines showed the transfer. Yellow arrows pointed to the transferred mitochondria.

(C) The representative images about mitochondria transfer between BMSCs and Müller cells (C1) or Müller-rot cells (C2) after direct coculture for 24 h. Yellow arrows pointed to the transferred mitochondria in Müller cells, showing the typical mitochondrial transfer ways as the figure A, B showed.

(D) Effect of rotenone treatment on the ratio of mitochondria transfer between BMSCs and MG. n⩾23.

Data are presented as the mean ± standard deviation (SD), ****P < 0.0001 (T-test for D). Scale bars: 20μm (A, B). 100μm (C).

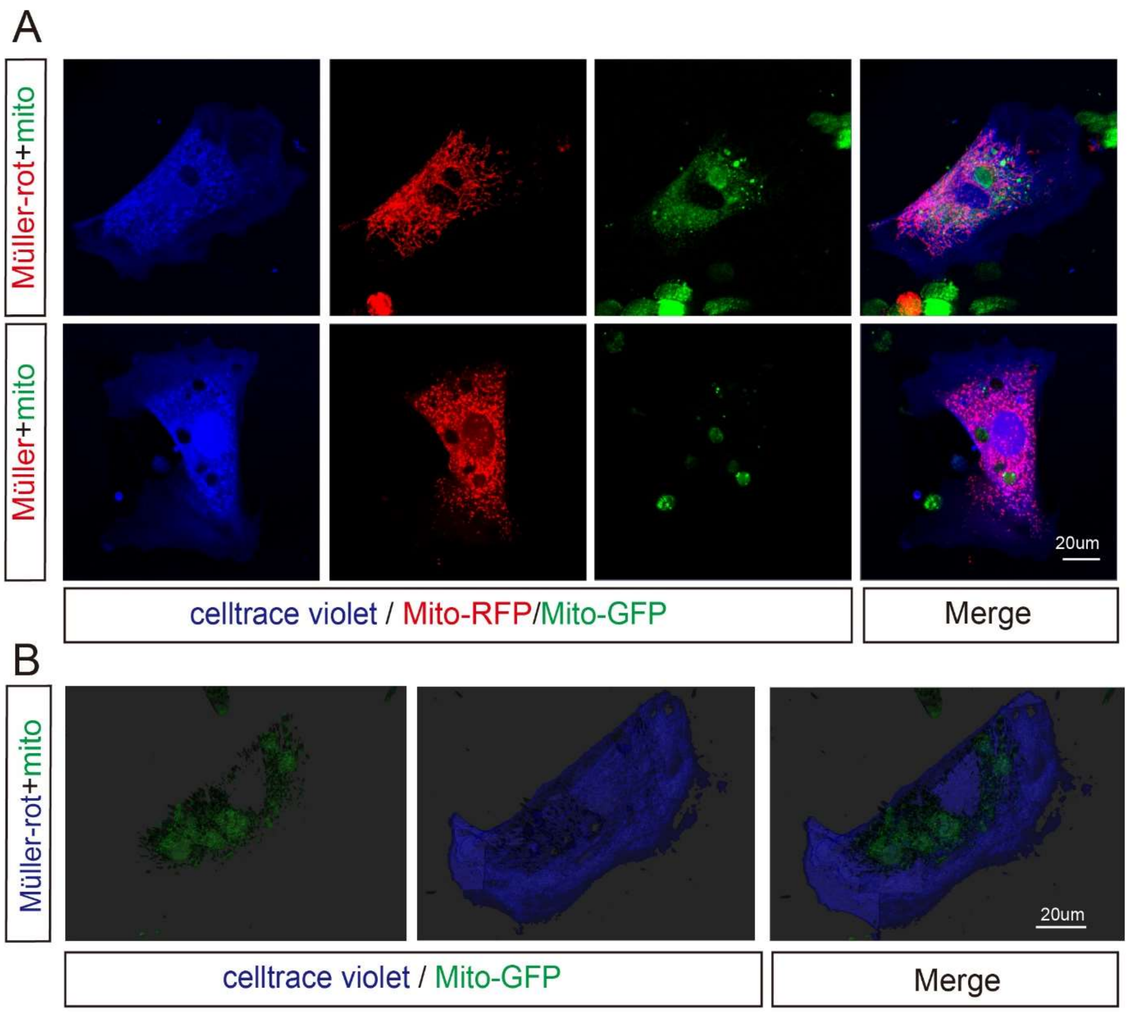

Figure S4. The treatment of rotenone enhanced the internalization of BMSCs-mito in Müller cells after co-culture for 24h, related to Figure 2.

(A) Celltrace violet-labelled(blue) the whole Müller cells, mitochondria of Müller cells labelled by Mito-RFP (red), BMSCs-mito labelled by Mito-GFP (green).

(B) Three-dimensional reconstruction of A.

Scale bars: 20μm (A).

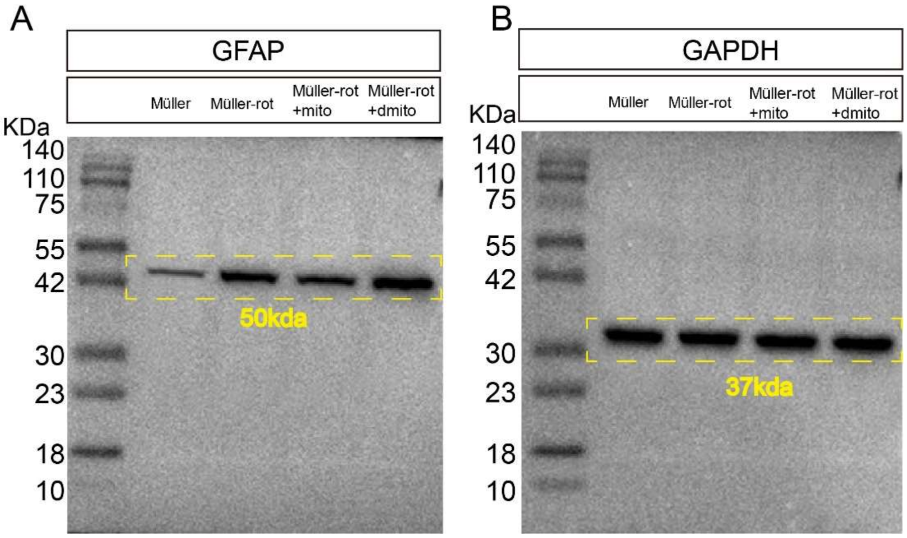

Figure S5. Intact images of Western blotting, related to Figure 4.

(A) The images of protein bands of GFAP.

(B) The images of protein bands the reference protein GAPDH**.**

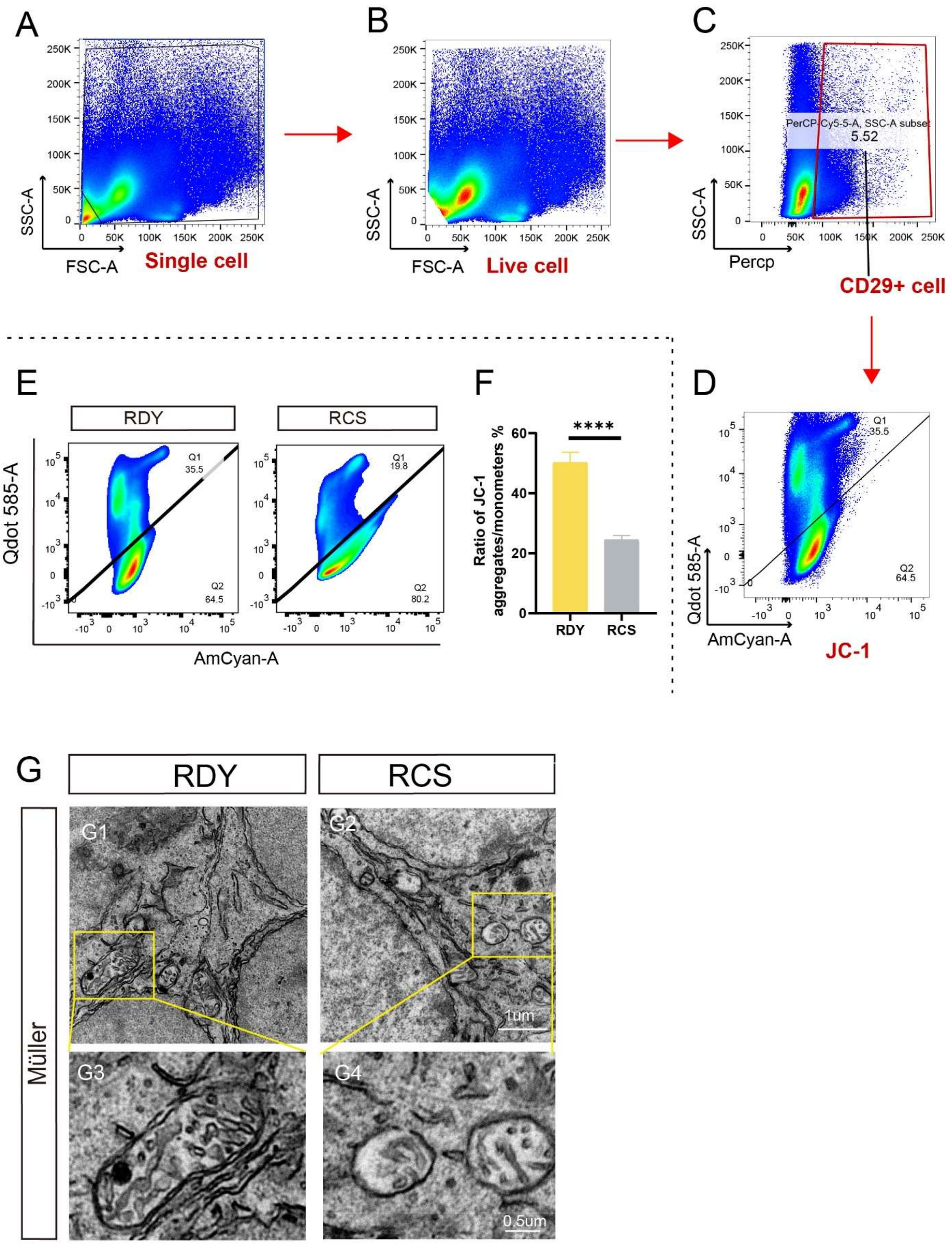

Figure S6. The Müller cells in the retinas of RCS rats exhibit mitochondrial dysfunction and morphological abnormalities, related to Figure 5 and STAR Methods.

(A-D) The representative analysis process of flow cytometry for mitochondrial membrane potential in retinal Müller cells of RCS rats or the normal groups RDY at 7 weeks after birth. A: single cells; B: live cells; C: $CD29^+$ cells, which labelled the Müller cells; D: Flow cytometry analysis of mitochondrial membrane potential (MMP) with JC-1 in Müller cells, Q1 represents JC-1 aggregates: normal mitochondria, Q2 represents JC-1 monomers: MMP decrease. Number below the Qn is the ratio of this part cells.

(E) Flow cytometry analysis of mitochondrial membrane potential (MMP) with JC-1 in Müller cells of RCS

rats or the normal groups RDY at 7 weeks after birth. while Q1(Qdot 585^{+}) represents JC-1 aggregates: higher MMP, Q2(Amcyan^{+}) represents JC-1 monomers: lower MMP. Number below Qn is the ratio of this part.

(F) Analysis of the ratio of MMP: JC-1 aggregates/JC-1 monomers. Higher ratio showed healthier mitochondrial function. n=4.

(G) The Transmission Electron Microscopy results of mitochondrial of Müller cells in the retina of RCS rat and the normal control rat RDY at 7w after birth. The morphological abnormalities occured in RCS rat retinal Müller cells.

Data are presented as the mean ± standard deviation (SD), ****P < 0.0001 (T-test for F). Scale bars: 10μm (G1, G2). 0.5μm (G3, G4).

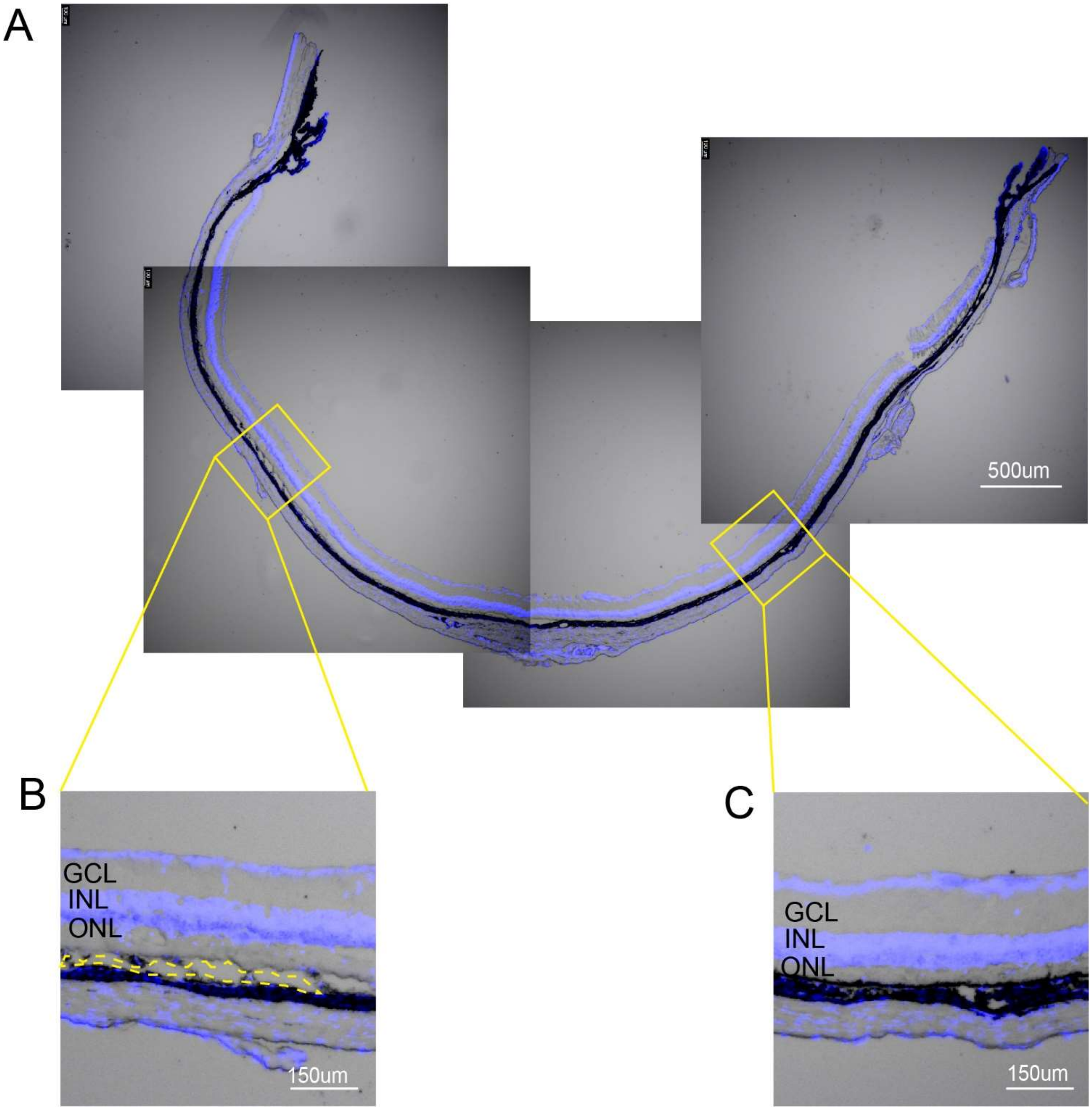

Figure S7. Puzzle pictures of whole retina of RCS rat after 6 weeks of subretinal transplantation of BMSCs-mito, related to Figure 5.

(A) Puzzle pictures of whole retina of RCS rat after 6 weeks of subretinal transplantation of BMSCs-mito. Transplant area retinal had detachment. No tumor formation. Merge pictures of DAPI (blue), white phase. Black area is RPE cells layer.

(B) Transplant area retinal showed some detachment. Ganglion cell layers (GCL), inner nuclear layers (INL), outer nuclear layers (ONL), yellow dashed line area is subretinal space (SRS).

(C) Opposite of the transplant area retinal showed little detachment.

Scale bars: 500µm (A), 150µm (B, C).

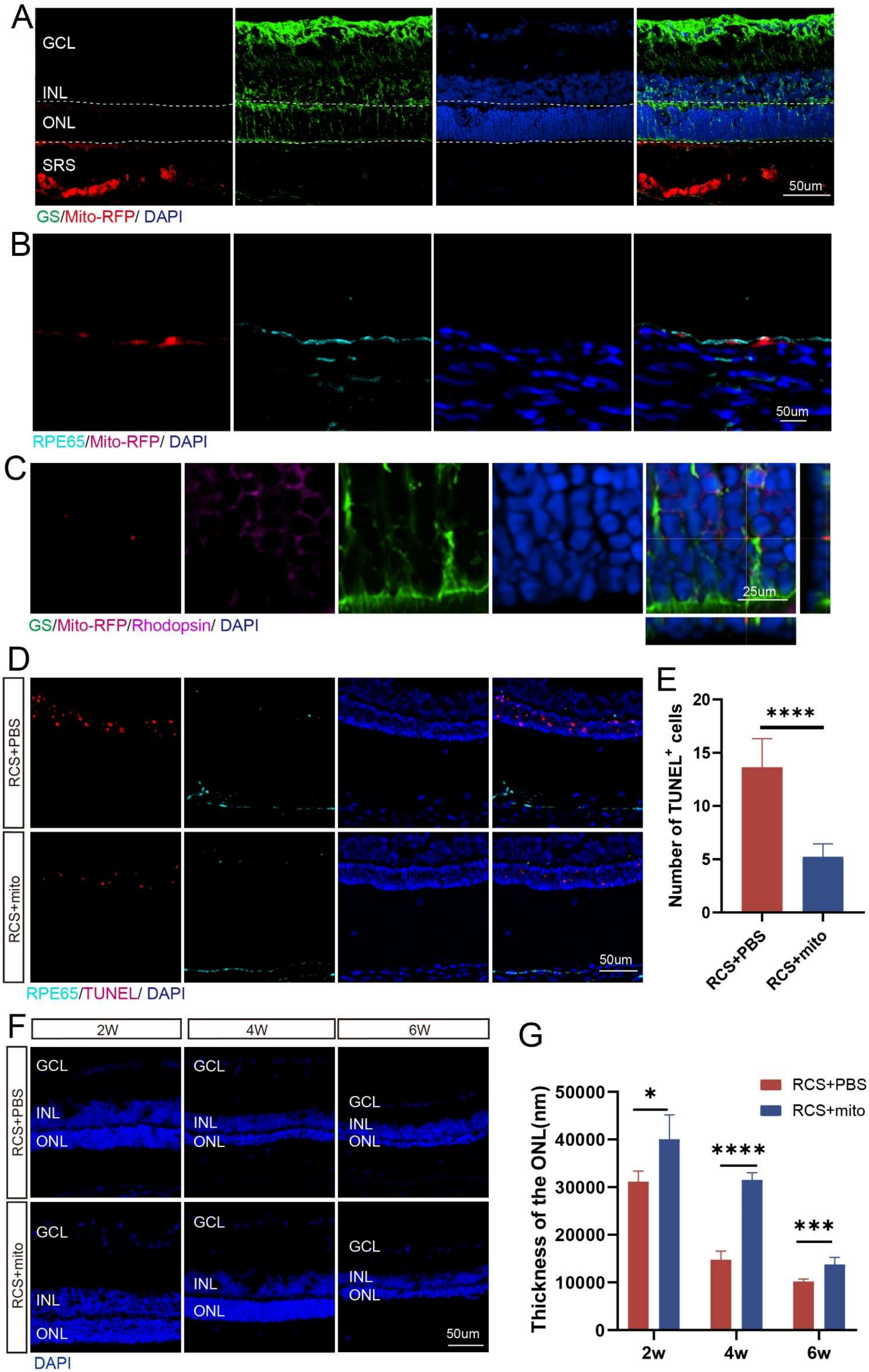

A
GCL
INL
ONL
SRS
50um
GS/Mito-RFP/ DAPI
B
50um
RPE65/Mito-RFP/ DAPI
C
25um
GS/Mito-RFP/Rhodopsin/ DAPI
D
RCS+PBS
RCS+mito
50um
RPE65/TUNEL/ DAPI
E
Number of TUNEL+ cells
20
15
10
5
0
****
RCS+PBS
RCS+mito
F
2W
4W
6W
GCL
INL
ONL
50um
DAPI
G
Thickness of the ONL(nm)
50000
40000
30000
20000
10000
0
*
****
***
RCS+PBS
RCS+mito
2w
4w
6w

Figure S8. The impact on retinal pigment epithelium (RPE) cells and photoreceptor cells of mitochondria of BMSCs in retina degenerated RCS rat after subretinal transplantation, **related to Figure 5.**

(A) Isolated Mitochondrion of BMSCs (BMSCs-mito) labelled by Mito-RFP (red), Müller cells staining by GS (green), DAPI (blue), 3 days after subretinal transplantation of mitochondria. Most of mitochondria located in the subretinal space. Ganglion cell layers (GCL), inner nuclear layers (INL), outer nuclear layers (ONL), subretinal space (SRS).

(B) BMSCs-mito labelled by Mito-RFP (red), RPE cells staining by RPE65 (cyan), DAPI (blue), 3 days after subretinal transplantation of mitochondria. Part of the BMSCs-mito located into the RPE cells.

(C) BMSCs-mito labelled by Mito-RFP (red), Müller cells staining by GS (green), photoreceptor cells staining by Rhodopsin (magenta), DAPI (blue), 3 days after subretinal transplantation of mitochondria. little of the BMSCs-mito located into the photoreceptor cells.

(D) Representative image of TUNEL of retina, 4 weeks after transplantation of rats in RCS+PBS groups and RCS+mito groups. $TUNEL^+$(red) labelled the apoptosis cells nuclei, RPE cells staining by RPE65 (cyan), DAPI (blue).

(E) Analysis of $TUNEL^+$ cells of retina. n=7.

(F) Representative image of the thicknesses of outer nuclear layers after BMSCs-mito transplantation. DAPI (blue).

(G) Analysis of the thicknesses of outer nuclear layers of retina. $n \geqslant 3$.

Data are presented as the mean ± standard deviation (SD), *P < 0.05, ***P < 0.001, ****P < 0.0001 (T-test for E, G). Scale bars: 50μm (A, B, D, F), 25μm (C).

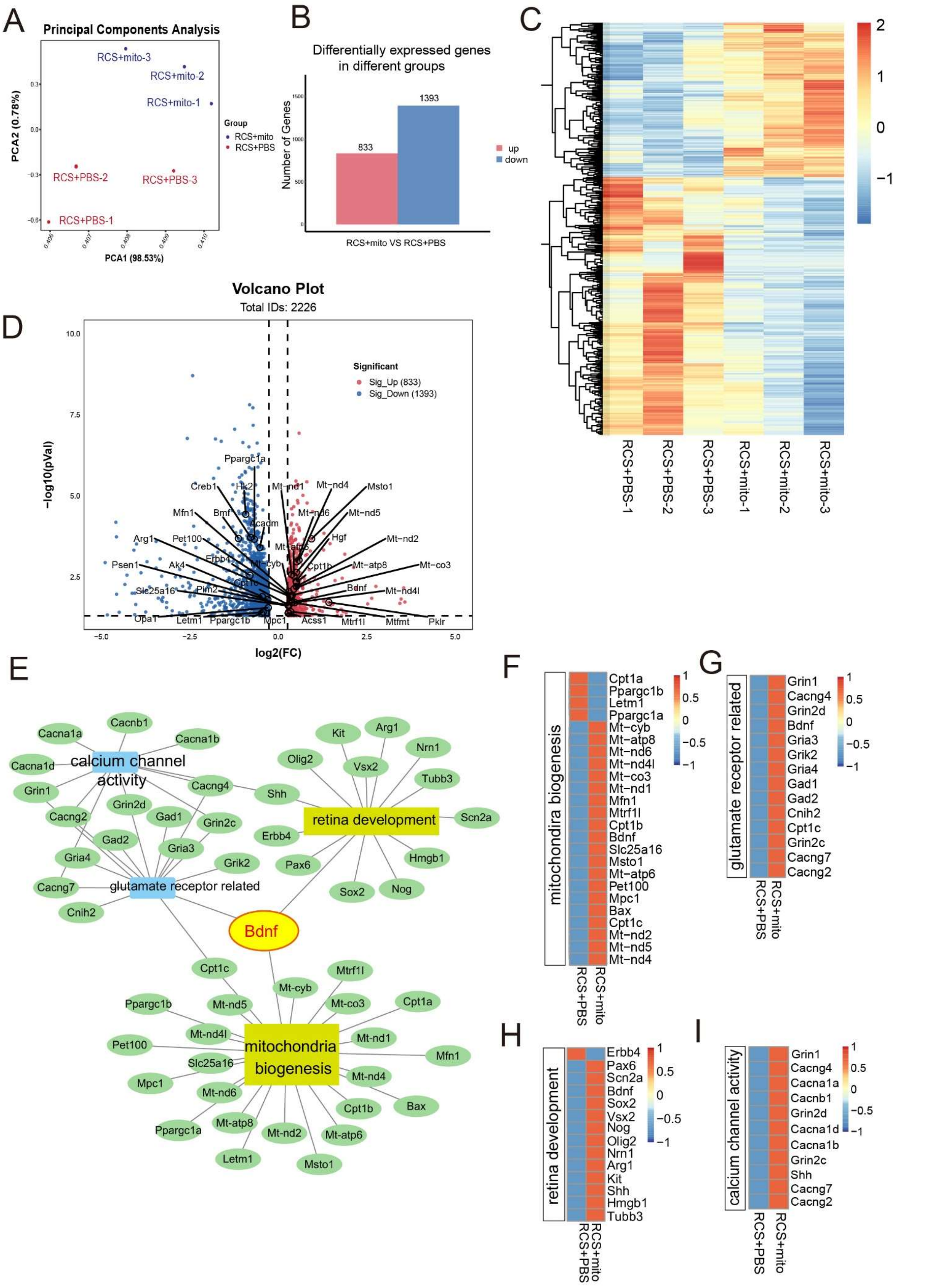

Figure S9. The pictures of PCA, DEGs, Volcano plot, and Cluster heat map of RCS+mito groups comparing to RCS+PBS groups in vivo, related to Figure 6.

(A) The principal components analysis (PCA) pictures between RCS+mito and RCS+PBS.

(B) The differentially expressed genes in different groups. FC > 1.2 and P < 0.05.Red is up, blue is down.

(C) The differentially expressed mRNAs in RCS+mito and RCS+PBS groups were classified and clustered. Each row represents a single mRNA, and each column represents one tissue sample. Red represented high relative expression; blue represented low relative expression.

(D) Volcano plot maps of DEGs. Mitochondrial genes showed gene name.

(E) Interaction diagram of GO pathway analysis with the differentially expressed genes. Bdnf played a key role.

(F-I) Heatmap of mitochondria biogenesis, glutamate receptor-related, retina development and calcium channel activity. Red is up, blue is down.

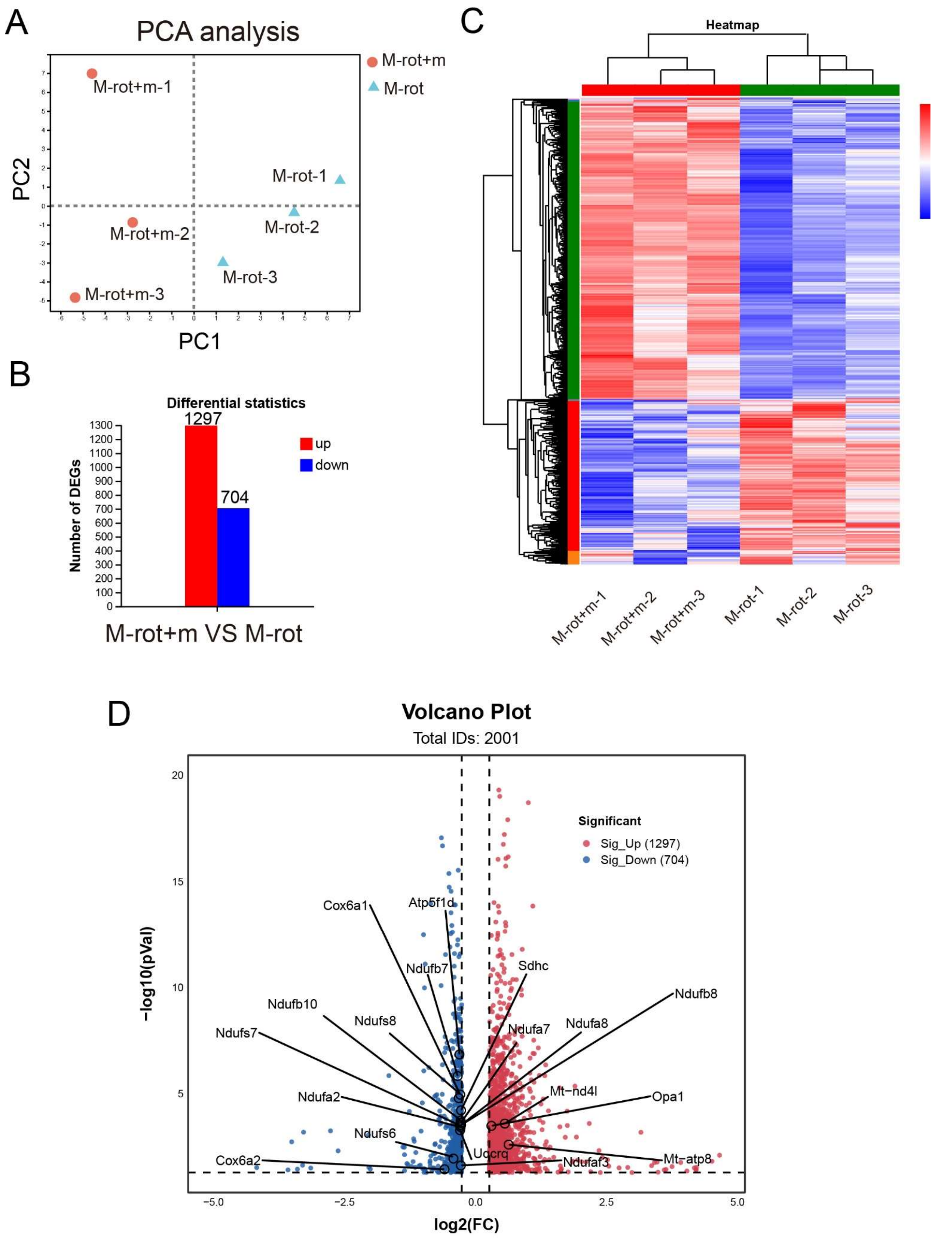

Figure S10. The pictures of PCA, DEGs, Volcano plot and Cluster heat map of Müller-rot+mito groups compared to Müller-rot groups in vitro, related to Figure 7.

(A) The principal components analysis (PCA) pictures between Müller-rot+mito (M-rot+m) and Müller-rot (M-rot).

(B) The differentially expressed genes in different groups at FC > 1.2 and $P < 0.05$. Red is up, blue is down.

(C) The differentially expressed mRNAs in Müller-rot+mito and Müller-rot groups were classified and clustered. Each row represents a single mRNA, and each column represents one tissue sample. Red represented high relative expression; blue represented low relative expression.

(D) Volcano plot maps of DEGs. Mitochondrial genes showed gene name.

Table S1. Summary of primary and secondary antibodies, related to STAR Methods.

Antibody name	Source	Catalog	Dilution ratio
Glial fibrillary protein (GFAP)	Abcam	AB7260	1:400 (IF); 1:1000 (WB)
Glutamine synthetase (GS)	Abclonal	A19641	1:400 (IF)
GAPDH	Immunoway	AC033	1:1000 (WB)
RPE65	Santa cruz	SC390787	1:400 (IF)
Rhodopsin	Abcam	AB98887	1:400 (IF)
Vimentin	Santa cruz	SC6260	1:400(IF)
488 donkey-mouse	Invitrogen	A21202	1:500
568 donkey-rabbit	Invitrogen	A10042	1:500
488 donkey-rabbit	Invitrogen	A21206	1:500
HRP-Goat anti-rabbit	Beyotime	A0208	1:2000
HRP-Goat anti-mouse	Beyotime	A0216	1:2000

Table S2. Primer sequences, related to STAR Methods.

Gene name	Forward 5’-3’	Reverse 5’-3’
Mt-DNA	GGTTCTTACTTCAGGGCCATCA	TGATTAGACCCGTTACCATCGA
Mt-nd1	TCACCCTAGTAGAACGGAAA	AATGGAGTAGACTGAAAGAC
Mt-nd4l	ATATTTCGCTCCCACTTAAT	GGCTAAACCTACTGCTGCTT
Mt-atp6	ATCTATTTGCCTCTTTCA	GTTCGTCCTTTTGGTGTG
Mt-cytb	CGAAACAGGATCAAATAA	GTAGAATAGCGTAGGCAA
Mt-cox3	ATTCTTCTTTGCCGGATTTT	GGTTTCGGTTGCCTTCTATT
Mfn1	GATGAGATTTGTCGCCTGTC	CATGCCATCTTCTATGTGCT
Cox6a1	GAGGGTTCAGCTCGTATTTGGA	GGATTGTGGAAGAGGGTATGGTTA
Ndufs7	CTACTCCTACTCGGTTGTTCGTGG	CAGCTTCTGTTCGCGTTTGATC
OPA1	TCTGCTGTTGGAGGTGGCTAT	TGTCCAGGTCAGGAGCGAAA
BDNF	GGATGAGGACCAGAAGGT	AGAAAGAGCAGAGGAGGC
Atp5mg	TTACTCGAAGCCTCGATT	TACCAGTTTGGGCACTGT
β-actin	GTTGACATCCGTAAAGACCT	CCACCAATCCACACAGAGTA
